# A prometabolite strategy inhibits cardiometabolic disease in an ApoE^–/–^ murine model of atherosclerosis

**DOI:** 10.1172/jci.insight.191090

**Published:** 2025-08-08

**Authors:** Taryn N. Beckman, Lisa R. Volpatti, Salvador Norton de Matos, Anna J. Slezak, Joseph W. Reda, Ada Weinstock, Leah Ziolkowski, Alex Turk, Erica Budina, Shijie Cao, Gustavo Borjas, Jung Woo Kwon, Orlando deLeon, Kirsten C. Refvik, Abigail L. Lauterbach, Suzana Gomes, Eugene B. Chang, Jeffrey A. Hubbell

**Affiliations:** 1 University of Chicago Pritzker School of Molecular Engineering, and; 2 Committee on Molecular Metabolism and Nutrition, University of Chicago, Chicago, Illinois, USA.; 3 Department of Biomedical Engineering, and; 4 Department of Chemical and Biological Engineering, Northwestern University, Evanston, Illinois, USA.; 5 Medical Scientist Training Program, Prtizker School of Medicine, and; 6 University of Chicago Pritzker School of Medicine, Department of Medicine, University of Chicago, Chicago, Illinois, USA.; 7 Department of Pharmaceutics, School of Pharmacy, University of Washington, Seattle, Washington, USA.; 8 Department of Pathology,; 9 Committee on Cancer Biology, and; 10 Committee on Immunology, University of Chicago, Chicago, Illinois, USA.; 11 Department of Chemical and Biomolecular Engineering,; 12 Department of Chemical and Biomolecular Engineering, Tandon School of Engineering, and; 13 Departments of Biology and Chemistry, Faculty of Arts & Science, New York University, New York, New York, USA.; 14 Department of Biochemistry and Molecular Pharmacology, NYU Langone Health, New York, New York, USA.

**Keywords:** Inflammation, Therapeutics, Atherosclerosis, Obesity, Transport

## Abstract

Butyrate, a microbiome-derived short-chain fatty acid with pleiotropic effects on inflammation and metabolism, has been shown to significantly reduce atherosclerotic lesions, rectify routine metabolic parameters such as low-density lipoprotein cholesterol (LDL-C), and reduce systemic inflammation in murine models of atherosclerosis. However, its foul odor, rapid metabolism in the gut and thus low systemic bioavailability limit its therapeutic effectiveness. Our laboratory has engineered an ester-linked L-serine conjugate to butyrate (SerBut) to mask its taste and odor and to coopt amino acid transporters in the gut to increase its systemic bioavailability, as determined by tissue measurements of free butyrate, produced by hydrolysis of SerBut. In an apolipoprotein E–knockout (ApoE)^–/–^ mouse model of atherosclerosis, SerBut reduced systemic LDL-C, proinflammatory cytokines, and circulating neutrophils. SerBut enhanced inhibition of plaque progression and reduced monocyte accumulation in the aorta compared with sodium butyrate. SerBut suppressed liver injury biomarkers alanine transaminase and aspartate aminotransferase and suppressed steatosis in the liver. SerBut overcomes several barriers to the translation of butyrate and shows superior promise in slowing atherosclerosis and liver injury compared with equidosed sodium butyrate.

## Abstract 2

A butyrate amino acid linkage, “SerBut,” has enhanced circulation by “hitching a ride” on intestinal transporters, enhancing efficacy compared to butyrate in cardiometabolic disease.

## Introduction

Atherosclerosis is a chronic immunometabolic disease that is a leading cause of cardiovascular disease (CVD) and death globally (1). It is associated with endothelial cell dysfunction, retention of cholesterol-rich lipoproteins in arterial walls, and chronic inflammation (2, 3). High plasma low-density lipoprotein–cholesterol (LDL-C) levels have historically been considered the most significant risk factor for promoting development and progression of atherosclerosis. LDL-lowering medications such as statins decreased CVD-related mortalities for the first decade of their use, but CVD mortality has steadily increased since 2014 with alarming rising rates in younger patients (4). Furthermore, statins are associated with serious side effects that can result in discontinuation, including liver toxicity. Patients with CVD often present with liver disease comorbidities, and liver injury and steatosis are strong predictors of early atherosclerosis (5, 6). Therefore, alternative medications are needed for these patients, including those with difficulty tolerating statins. More recently, PCSK9 inhibitors have been approved to lower LDL-C levels but are limited by recurrent intravenous injections and resulting immunogenicity (7, 8).

While statins remain the gold standard for atherosclerosis therapy, decades of research have revealed the critical role of the immune system in disease progression. Following initial lipoprotein deposition and oxidation in the subendothelial space, tissue-resident immune cells become activated, and monocytes are recruited to the early lesions (9). Oxidized LDL can activate the NF-κB pathway in recruited monocytes, resulting in the upregulation of proinflammatory genes, such as adhesion molecules, chemokines, and cytokines (e.g., IL-1α/β, IL-6, and IL-12) (10). For example, monocyte-chemoattractant protein 1 (MCP-1), also known as C-motif chemokine ligand 2 (CCL2), regulates inflammatory monocyte trafficking between the bone marrow, circulation, and atherosclerotic plaques by binding to its cognate receptor CCR2 on immune cells and endothelial cells. Upon uptake of excess oxidized LDL, monocytes differentiate into lipid-laden foam cells, which become dysfunctional and contribute to the formation of necrotic core in later stages of plaque development (9).

Several studies have reported a predictive relationship between biomarkers of inflammation (e.g., C-reactive protein) and cardiovascular events, as well as a direct benefit of antiinflammatory interventions in humans (11–15). For example, colchicine, which inhibits the NF-κB–regulated NLR family pyrin-domain-containing 3 (NLRP3) inflammasome, provided proof of concept of the benefits of broad immunosuppression (11, 12). Colchicine treatment reduced cardiovascular events but was associated with increased lethal infections at high doses, although it received FDA approval at low doses (15–18). Antibody-mediated neutralization of IL-1β has also been evaluated in clinical trials but did not meet the primary end point at low doses and was similarly associated with a higher incidence of lethal infections (14). Thus, there is a need for innovative therapeutics that lower lipids and lipid-driven inflammation without inducing liver toxicity or impairing the patient’s ability to fight common infections.

Partially due to its role in regulating inflammation, the gut-heart axis has been repeatedly implicated in CVD (19, 20). High-fat diets (HFDs) lead to microbiome dysbiosis and increased risk of CVD, while high-fiber diets lead to increased production of short-chain fatty acids (SCFAs) and atheroprotection (19, 21–23). Butyrate, an antiinflammatory SCFA and fermentation product of the gut microbiome, has received widespread attention for its immunomodulatory applications in immunometabolic diseases such as obesity, atherosclerosis, and nonalcoholic fatty liver disease (24–29). Butyrate exerts antiinflammatory effects in immune cells through inhibition of histone deacetylases (HDACs), resulting in suppression of NF-κB activation and reduced proinflammatory cytokine secretion (24, 30)^.^ Other antiinflammatory mechanisms of butyrate are indirect, such as enhancing the intestinal barrier and thus decreasing the translocation of inflammatory molecules like lipopolysaccharide (LPS) (31). Butyrate is further proposed to be atheroprotective via regulation of lipid and glucose metabolism genes and improved gut microbiota diversity (31–33). In the liver, butyrate reduces hepatic steatosis by increasing fatty acid β-oxidation capacity and decreasing lipid synthesis (34). Many studies in experimental CVD animal models and humans have demonstrated the use of oral butyrate supplementation as both antiinflammatory and lipid lowering, underscoring the therapeutic potential of butyrate to influence development and progression of CVD and expand non-statin therapeutic options (32, 33, 35–38).

Despite this potential, pharmacological aspects of butyrate, whether administered orally or generated by fermentation in the gut, present substantial limitations. The high concentrations of systemic, circulating butyrate required for its antiinflammatory activity (e.g., HDAC inhibition) are not found at physiological levels (31, 39). Likewise, oral administration of exogenous butyrate is limited by its fast metabolism in the gut, resulting in low absorption into circulation (~3–4 mM in plasma), its foul odor and taste, and its large doses required. We have previously engineered a small molecule prometabolite consisting of butyrate esterified to L-serine, *O*-butyryl-L-serine (SerBut), to address the challenges of butyrate translation (28). We hypothesize that amino acid transporters in the small intestine allow SerBut to exit the lumen in the small bowel and thus bypass metabolism in the large bowel, increasing systemic bioavailability. This esterification favorably masks the odor and taste, increases half-life, and maintains the pleiotropic benefits of native butyrate upon hydrolysis.

Our group has shown that once- to twice-daily oral gavage of SerBut suppresses disease progression in murine models of autoimmune disease such as rheumatoid arthritis and multiple sclerosis (28). Here, we aimed to investigate the efficacy of SerBut dosed through drinking water in a murine apolipoprotein E–knockout (ApoE^–/–^) model of atherosclerosis, examining effects on vascular inflammation, plaque formation and composition, systemic metabolic regulation, and HFD-induced liver injury. We show that this systemically bioavailable form of butyrate reduces plaque in the aortic root via inhibition of progression, accumulation of immune cells in the aorta, and liver steatosis. Furthermore, plasma analysis shows SerBut reduces the fraction of LDL-C, systemic inflammatory cytokines, circulating neutrophils, and liver damage markers elevated by HFD feeding. Under these experimental conditions, equidosed treatment with sodium butyrate (NaBut) failed to produce these favorable effects on cardiovascular or hepatic(immuno)pathology.

## Results

### SerBut is a cytocompatible and stable carrier of antiinflammatory free butyrate.

**Figure 1:**
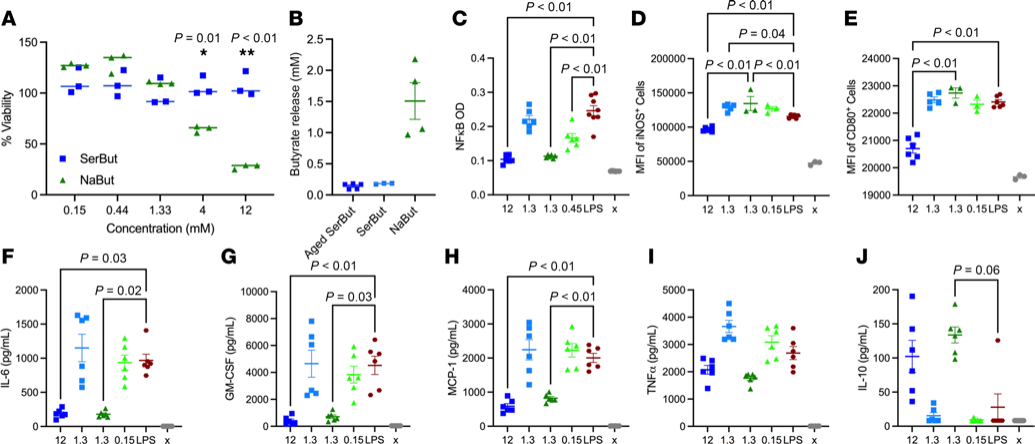
SerBut reduces cytotoxicity as a carrier of inflammatory NF-κB pathway–suppressing butyrate in vitro. (**A**) Viability of RAW 264.7 cells treated with 0.15, 0.44, 1.33, 4, and 12 mM SerBut or NaBut for 24 hours. (**B**) LC-MS/MS analysis of free butyrate in solution of aged SerBut (incubated at 37°C in FBS-supplemented media for 1 month) and freshly dissolved SerBut and NaBut. (**C**) NF-κB activity of RAW Blue reporter cells upon treatment with the indicated concentrations of SerBut or NaBut for 24 hours followed by stimulation with 200 ng/mL LPS for 6 hours. (**D**–**J**) RAW 264.7 cells treated for 24 hours with the indicated concentrations of SerBut or NaBut followed by stimulation with 200 ng/mL LPS for 6 hours. (**D**–**E**) Mean fluorescence intensity (MFI) of iNOS^+^ or CD80^+^ RAW 264.7 cells analyzed by flow cytometry. (**F**–**J**) Cytokine concentrations in the cell culture supernatant of RAW 264.7 cells. “x” indicates untreated group. In **A**, *n* = 3 per group representing technical replicates. In **B**, *n* = 3 freshly prepared samples per 2 distinct experiments pooled for aged SerBut and NaBut groups, while fresh the SerBut group represents a single experiment. One sample in the aged SerBut group and 2 samples in the NaBut group were lost due to filtering malfunctions or LC-MS/MS autosampling malfunctions. In **C**, *n* = 6 technical replicates. Data represent mean ± SEM. Experiments were performed 2 or more times with similar results. In **A**, 2-way ANOVA was performed. Statistical analyses were performed using a 1-way ANOVA with Tukey’s, Welch’s (if standard deviations were significantly different by Bartlett and Brown-Forsyth tests), or Kruskal-Wallis (if data were not normally distributed determined by Shapiro-Wilk test) post hoc test in **C**–**J**. *P* values less than 0.10 are shown.

Previously, our laboratory has shown that SerBut suppressed inflammatory marker expression in bone marrow–derived dendritic cells (BMDCs) (28, 40). Importantly, we additionally observed that SerBut maintained attenuated HDAC inhibition activity in a macrophage-like cell line (RAW 264.7) (28). We hypothesized that SerBut could be dosed much higher than 0.2 mM, the previously administered dose, without inducing cytotoxicity. Thus, we evaluated the cytocompatibility of SerBut compared to NaBut in RAW 264.7 macrophages (Figure 1A and Supplemental Figure 1; supplemental material available online with this article; https://doi.org/10.1172/jci.insight.191090DS1). While NaBut significantly reduced cell viability at 4 mM and above, SerBut exhibited no cytotoxicity even at a concentration of 12 mM. We hypothesized this reduction in cytotoxicity, and the previously noted attenuation of HDAC inhibition activity, was due to inactivation of the butyrate upon conjugation to serine. Using liquid chromatography with tandem mass spectrometry (LC-MS/MS), we determined that approximately 10% of the SerBut sample was free butyrate at the time of measurement (Figure 1B). To investigate stability, we repeated the experiment with SerBut in FBS-supplemented complete media stored for 1 month at 37°C (Figure 1B). The relative concentration of free butyrate did not change, indicating that esterases in FBS did not hydrolyze SerBut overtime but some free butyrate was available in the solution.

Exogenous NaBut supplementation is known to exert antiinflammatory effects through NF-κB suppression in macrophages in vitro and in vivo (24, 30, 35). We next investigated whether SerBut maintained NaBut’s antiinflammatory effects in macrophage-like RAW 264.7 cells. SerBut at 12 mM significantly suppressed LPS-induced NF-κB activation comparable to 1.3 mM NaBut (Figure 1C), aligning with our finding that 10% of the SerBut sample was free butyrate (Figure 1B). NF-κB activation regulates the inflammatory macrophage phenotype, inducing proinflammatory cytokine secretion and reactive oxygen species production. Therefore, we next investigated expression of activation and inflammation markers downstream of NF-κB inhibition. Interestingly, only 12 mM SerBut significantly reduced both induced nitric oxide synthase (iNOS) and CD80 expression after LPS stimulation (Figure 1, D and E). Furthermore, 12 mM SerBut suppressed NF-κB–regulated production of proinflammatory cytokines that are known to orchestrate inflammatory immune cell activation and recruitment, IL-6, MCP-1, and GM-CSF, as effectively as 1.3 mM NaBut (Figure 1, F–J). The antiinflammatory cytokine IL-10 was significantly increased by NaBut treatment, while SerBut treatment produced a trending increase compared with LPS-stimulated controls (Figure 1J). The iNOS-suppressing capacity of high doses of SerBut was maintained even during longer LPS stimulations (Supplemental Figure 1C) and had beneficial effect on activation markers CD80 and CD86 compared with NaBut but not LPS alone (Supplemental Figure 1, A and B). However, this effect was not observed when butyrate was dosed after LPS stimulation (Supplemental Figure 1, D–F).

### Seryl modification of butyrate significantly enhances its bioavailability and therapeutic efficacy in atherosclerotic mice.

**Figure 2:**
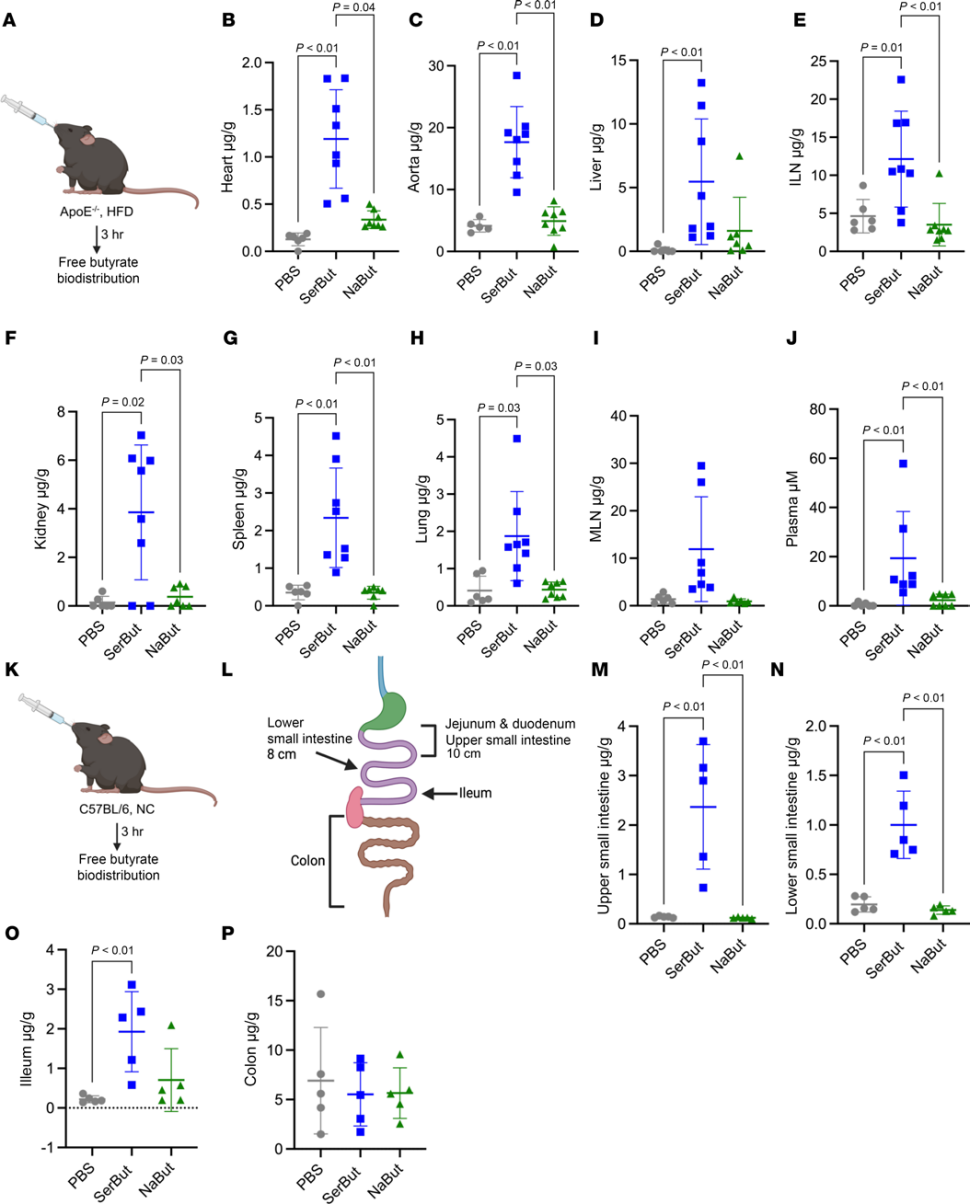
Seryl modification increases oral bioavailability of butyrate in atherosclerotic organs. (**A**) Experimental schema of ApoE^–/–^ mice fed an HFD for 8 weeks, after which they received a single equimolar gavage of 272 mM butyrate as SerBut or NaBut. After 3 hours, mice were perfused, and the amount of butyrate per gram of tissue was measured in the (**B**) heart, (**C**) aorta, (**D**) liver, (**E**) inguinal lymph nodes (ILNs), (**F**) kidney, (**G**) spleen, (**H**) lung, (**I**) mesenteric lymph nodes (MLNs), and (**J**) plasma (μM). *n* = 8 mice in SerBut and NaBut groups. *n* = 6 mice in PBS group. (**K**) Experimental schema of C57BL/6 mice fed a normal chow (NC) diet, after which they received a single equimolar gavage of 272 mM butyrate as SerBut or NaBut. (**L**) Diagram of gut dissection. (**M**) Upper small intestine. (**N**) Lower small intestine. (**O**) Ileum. (**P**) Colon. Quantification was performed by LC-MS/MS upon derivatization with 3-nitrophenylhydrazine. Free butyrate (g) was normalized to tissue weight (g). Data points represent individual mice displayed with median ± SEM. Statistical analyses were performed using a 1-way ANOVA with Tukey’s, Welch’s (if SDs were significantly different by Bartlett and Brown-Forsyth tests), or Kruskal-Wallis (if data were not normally distributed determined by Shapiro-Wilk test) post hoc test. *P* values less than 0.10 are shown.

To determine the bioavailability of SerBut in cardiovascular organs of hyperlipidemic mice, we gavaged equimolar amounts of SerBut and NaBut to ApoE^–/–^ mice on an HFD (42% calories from fat) to ensure comparable consumption of butyrate by all mice at the given time point. Three hours after administration, we analyzed the biodistribution of free butyrate via LC-MS/MS. (Figure 2A). SerBut dosing significantly increased free butyrate in the heart and aorta compared with both NaBut-treated and PBS-treated mice (Figure 2, B and C). Corroborating our previous work (28), we also observed significant increases in the liver, inguinal lymph nodes (ILNs), kidney, spleen, lung, and plasma (Figure 2, D–H, and J). We also saw trending increases of free butyrate in the mesenteric lymph nodes (MLNs) of SerBut-treated mice (Figure 2I).

NaBut is limited in its translation by butyrate’s rapid consumption by colonocytes as a primary energy source in the distal gut. We propose that the enhanced biodistribution of free, active butyrate (following cleavage from serine) in SerBut-treated mice is due to small intestinal escape from the gut via amino acid transporters. To probe this hypothesis, we repeated the experiment and analyzed the biodistribution of free active butyrate in distinct sections of the intestinal tissue and the intestinal contents (Figure 2, K and L, and Supplemental Figure 2). Because neither ApoE^–/–^ nor HFD feeding would be expected to change amino acid receptor expression or function in the intestine, this was performed in C57BL/6 mice on a normal diet. There were no significant changes in free butyrate in the intestinal contents for SerBut-treated mice (Supplemental Figure 2). Conversely, in the upper and lower small intestine and ileum tissue (41), we observed significantly more free, active butyrate in SerBut-treated mice compared with both PBS- and NaBut-treated mice, indicating enhanced SerBut transport out of the gut in the small intestine (Figure 2, M–O). We saw no increase in butyrate in the colon tissue (Figure 2P).

**Figure 3:**
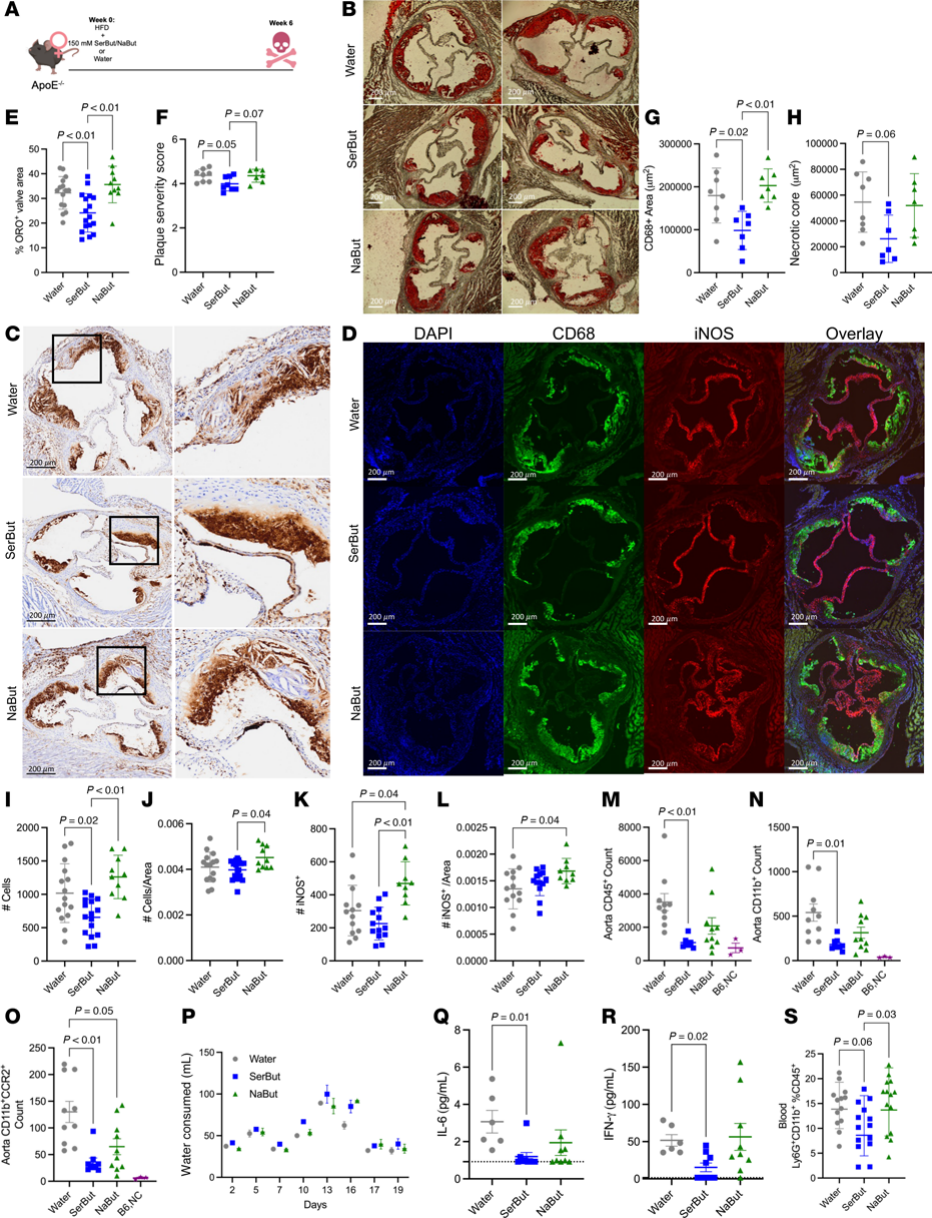
SerBut suppresses the progression of atherosclerotic plaque, accumulation of immune cells in the aorta, and systemic inflammation. (**A**) Schema of female ApoE^–/–^ mice on an HFD with 150 mM SerBut or NaBut on a butyrate basis, or water ad libitum for 6 weeks. (**B**) Oil Red O (ORO) staining of aortic root plaque. (**C**) CD68^+^ IHC staining of aortic root plaque. Original magnification, ×20. (**D**) Representative images of immunofluorescent staining of aortic root. (**E**) Quantification of ORO^+^ aortic root valve area. (**F**) Histological Stary scoring of plaque severity, (**G**) quantified CD68^+^ area, and (**H**) necrotic core of CD68^+^ IHC staining. (**I**) Quantified DAPI^+^, (**J**) DAPI^+^ per plaque area, (**K**) iNOS^+^, and (**L**) iNOS^+^ cells per plaque area in immunofluorescence stain. (**M**–**O**) Flow cytometry of immune cells infiltrating the aorta: (**M**) CD45^+^ total leukocytes, (**N**) CD11b^+^ monocytes, and (**O**) CCR2^+^CD11b^+^ monocytes. (**P**) Water consumed by cages for the first 19 days of experiment. Data represent mean water consumed between 2 cages (replicates) as weight change of bottle converted to water volume. Error bars represent SD. (**Q**) Plasma IL-6 and (**R**) IFN-γ. (**S**) Blood flow cytometry of Ly6G^+^CD11b^+^ circulating neutrophils. *n* = 5/cage and *n* = 10 mice per group. Data points represent individual mice displayed with median ± SEM. Statistical analyses were performed using a 1-way ANOVA with Tukey’s, Welch’s (if SDs were significantly different by Bartlett and Brown-Forsyth tests), or Kruskal-Wallis (if data were not normally distributed determined by Shapiro-Wilk test) post hoc test. *P* values less than 0.10 are shown. Outliers were removed by ROUT testing at *Q* = 1% in **I**–**L**. Scoring in **C** was done blinded. Data in **C** and **F**–**H** represent 2 independent pooled experiments: *n* = 10 for water and SerBut groups and *n* = 10 for NaBut groups in a single experiment. “B6,NC” denotes age-matched C57BL/6 mice on normal diet as non-statistical comparison to visualize healthy examples. Scale bars: 200 μm.

To investigate SerBut’s effect on cardiovascular disease, ApoE^–/–^ mice were given 150 mM SerBut or NaBut dissolved in water ad libitum over 6 weeks in concert with an HFD (Figure 3A). This study furthermore allowed us to investigate a more translatable method of administration by adding SerBut to the drinking water, in contrast with administering by oral gavage. Treatment with serine showed no significant differences from controls in murine models of autoimmune disease (28) and thus was not included in this study. After 6 weeks, we observed significantly less Oil Red O–stained plaque area in the aortic root of SerBut-treated mice compared with both control and NaBut-treated groups (Figure 3, B, C, and E). We then asked whether plaque complexity and stage were changed between treatment groups, potentially indicating suppression of disease progression. Utilizing Stary histological grading (42) and quantification of plaque components, we found a significant reduction in plaque severity score, CD68^+^ stained area, and necrotic core area in SerBut-treated mice compared with water-treated mice (Figure 3, F–H, and Supplemental Figure 3, A–D) and trended lower compared with NaBut-treated mice (Figure 3D). We then investigated the plaque immune phenotype by immunofluorescent staining of CD68, iNOS, and DAPI. Although the total numbers of CD68^+^ macrophages were reduced compared to NaBut-treated groups, the macrophages per unit area were not (Supplemental Figure 3F-I). However, both CD68^+^ cells and iNOS^+^ cells per unit area atherosclerotic lesion were increased in NaBut-treated groups, indicating a more inflamed plaque phenotype in NaBut-treated groups compared with both water-and SerBut-treated groups (Figure 3, I–L).

Since immune cell infiltration into the aorta has been shown to accelerate disease pathogenesis, we next asked whether SerBut reduced vascular inflammation (43). Through flow cytometry of the aorta, we found that only SerBut significantly reduced CD45^+^ total leukocytes, CD11b^+^ monocytes, and CCR2^+^CD11b^+^ monocytes in the aorta compared with control groups, while NaBut did not (Figure 3, M–O). CD11c^+^MHCII^hi^ dendritic cells, CD11b^+^F480^+^ macrophages, and CD19^+^ B cells were significantly reduced by both NaBut and SerBut compared with water controls (Supplemental Figure 4). Additionally, CD11b^+^Ly6C^hi^ monocytes and CD4^+^ T cells were significantly reduced only in SerBut-treated mice compared to water-treated controls (Supplemental Figure 4).

We next evaluated the effect of SerBut on systemic markers of inflammation. SerBut reduced the inflammatory cytokines IL-6 and IFN-γ in plasma significantly compared with control groups, while NaBut treatment did not (Figure 3, M and N). Both NaBut and SerBut showed a trend toward reduction in plasma IL-1β (Supplemental Figure 4). SerBut significantly reduced the frequency of circulating Ly6G^+^CD11b^+^ neutrophils compared with both NaBut- and water-treated controls (Figure 3S). Additionally, SerBut expanded regulatory T cells (Tregs) in the MLNs but not the skin-draining lymph nodes or spleen (Supplemental Figure 5). However, in both the MLNs and spleen SerBut reduced the Th17/Treg cell ratio, indicating a more tolerogenic phenotype. In the spleen, NaBut-treated mice also showed a lower Th17/Treg cell ratio (Supplemental Figure 5). In the MLNs, but not the ILNs, CD11b^+^F480^+^ macrophages exhibited significantly decreased inflammatory marker iNOS^+^ compared with controls, but not NaBut-treated mice (Supplemental Figure 6). Importantly, all cages consumed comparable amounts of water (Figure 3P), suggesting that the effect of SerBut is due to its immunological effects and not to the administration of a higher dose. Overall, SerBut exerted more efficacious antiatherogenic and antiinflammatory effects in vivo than NaBut systemically and at the site of disease compared with water-treated control groups.

Butyrate dosing has been shown to significantly affect the gut microbiome and its metabolic products (31, 44). Thus, we wanted to understand how the 2 types of exogenous butyrate (SerBut and NaBut) affected the microbiome, particularly the colonic microbiota. Based on our biodistribution data, we expected that SerBut is predominantly absorbed in the small bowel and transited to systemic circulation, thus avoiding the microbial communities found in the colon and their interaction with the host there. To understand the potential association between the microbiome and the antiatherosclerotic effects of SerBut and NaBut, we performed sequential multiple linear regressions on 16S rRNA sequencing of the colonic contents after treatment on HFD for 6 weeks (Supplemental Figures 7–9). We asked whether pathology readouts were associated with butyrate treatment (combining SerBut and NaBut together), seryl modification of butyrate, or the relative abundance of individual bacterial genera to identify specific taxa implicated in disease pathology. We found that seryl modification of butyrate, i.e., SerBut treatment, could alone explain the reduction in plaque after controlling for most bacterial genera. We asked whether the bacterial genera that were required to explain the reduction in the plaque were expanded by SerBut treatment, potentially indicating their mediation of SerBut’s beneficial effects (Supplemental Figure 7, B–G). None of these bacteria were expanded by SerBut treatment, indicating that they likely do not mediate SerBut’s beneficial effects on atherogenesis. The genus *Lactobacillus* was the only bacterial variable that alone could explain the reduction in plaque in the aortic root after controlling for the aforementioned variables (Supplemental Figure 8).

By contrast, unmodified-butyrate treatment could not explain the observed reduced disease pathology and required the abundance of multiple bacterial taxa (Supplemental Figure 9) to explain correlations with disease pathology. We then asked whether SerBut’s efficacy can be explained by expanding the abundance of *Lactobacillus* and found that both water control and NaBut groups had significantly more *Lactobacillus* than SerBut groups (Supplemental Figure 8B). Next, we asked whether the treatment group significantly modulated the relationship of *Lactobacillus* abundance with plaque (Supplemental Figure 8C). SerBut-treated groups showed no relationship between *Lactobacillus* and plaque, while both NaBut- and water-treated groups showed a negative correlation between *Lactobacillus* and plaque. Together, these data demonstrate the differences between the microbiome effects of these 2 forms of exogenous butyrate, suggesting differing mechanisms of action. We hypothesize that the majority of SerBut’s effect on plaque formation and inflammation may not be mediated though modulation of relative abundance of bacterial genera in the microbiome, although NaBut treatment may mediate some efficacy through bacterial expansion. Further sequencing may be able to provide species-level characterization that could identify single bacteria with differentially abundant growth or function due to treatment groups.

### SerBut suppresses HFD-induced liver injury.

**Figure 4:**
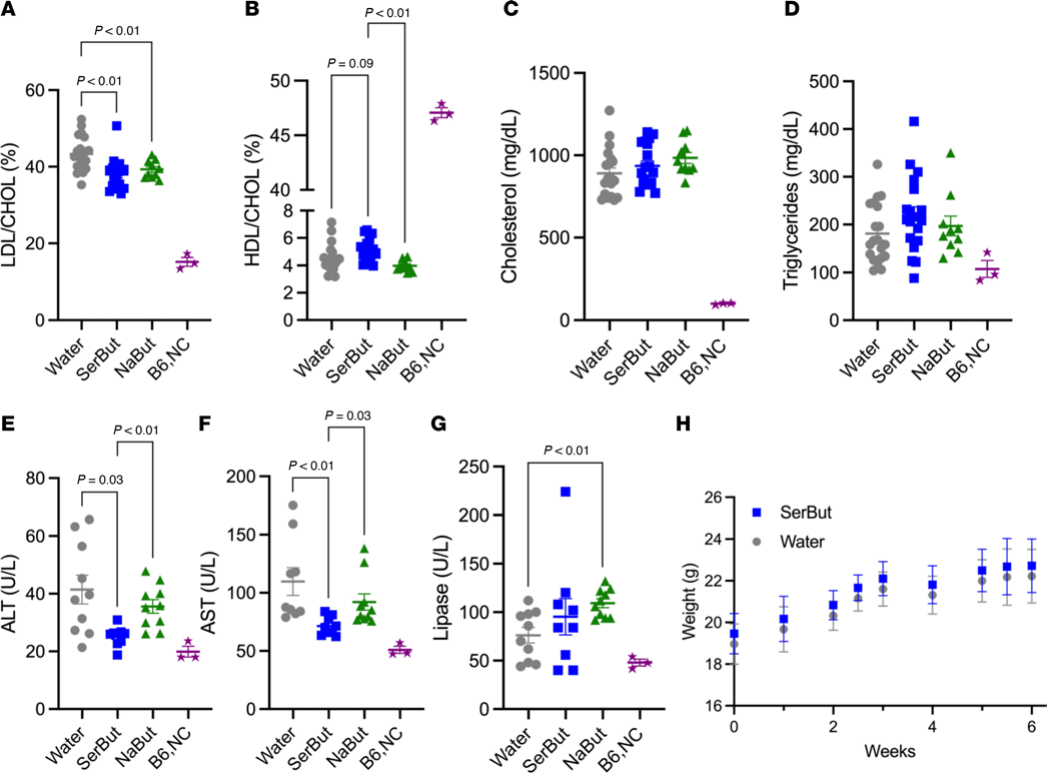
SerBut suppresses plasma LDL-C and reduces liver damage markers. Plasma taken at 6 weeks of treatment in the regimen described in Figure 3A. (**A**–**D**) Blood chemical analysis pooled between 2 duplicate studies: (**A**) Percentage LDL/total cholesterol, (**B**) percentage HDL/total cholesterol, (**C**) total cholesterol, and (**D**) triglycerides. (**E**–**G**) Blood chemical analysis: (**E**) alanine transaminase (ALT), (**F**) aspartate aminotransferase (AST), and (**G**) lipase. *P* values less than 0.10 are shown. (**H**) Average weight of mice in water- and SerBut-treated groups. “B6,NC” denotes 3 age-matched C57BL/6 mice on a normal diet as a non-statistical comparison to visualize healthy examples. Data points represent individual mice displayed with median ± SEM. Data in **A**–**D** represent 2 independent pooled experiments: *n* = 10 per group per experiment for water- and SerBut-treated groups; *n* = 10 in a single experiment for NaBut-treated group. Statistical analyses were performed using a 1-way ANOVA with Tukey’s, Welch’s (if SDs were significantly different by Bartlett and Brown-Forsyth tests), or Kruskal-Wallis (if data were not normally distributed determined by Shapiro-Wilk test) post hoc test. *P* values less than 0.10 are shown.

Since NaBut has been shown to reduce plasma lipids in murine models of atherosclerosis (38), we asked whether SerBut was also able to influence plasma lipids. LDL-C as a fraction of total cholesterol was significantly reduced by both SerBut and NaBut treatment (Figure 4A and Supplemental Figure 10). Additionally, the ratio of high-density lipoprotein (HDL) to total cholesterol was significantly increased in SerBut-treated mice compared with NaBut treatment (Figure 4B and Supplemental Figure 10). Neither SerBut nor NaBut influenced total cholesterol or triglycerides in the plasma (Figure 4, C and D). Interestingly, SerBut significantly reduced plasma alanine transaminase (ALT) and aspartate aminotransferase (AST) compared with both water control and NaBut groups (Figure 4, E and F), showing reduction in liver damage induced by HFD for SerBut but not NaBut treatment. NaBut-treated mice experienced a significant increase in lipase — a common marker of pancreatitis — in the plasma, while SerBut showed no effect (Figure 4G), suggesting that SerBut may have an enhanced safety profile compared with NaBut. The positive effects of SerBut were independent of body weight, as SerBut-treated mice had similar weights to water controls throughout the study (Figure 4H). Overall, seryl esterification significantly enhances the antiatherosclerotic hepatoprotective activity and safety profile of butyrate.

**Figure 5:**
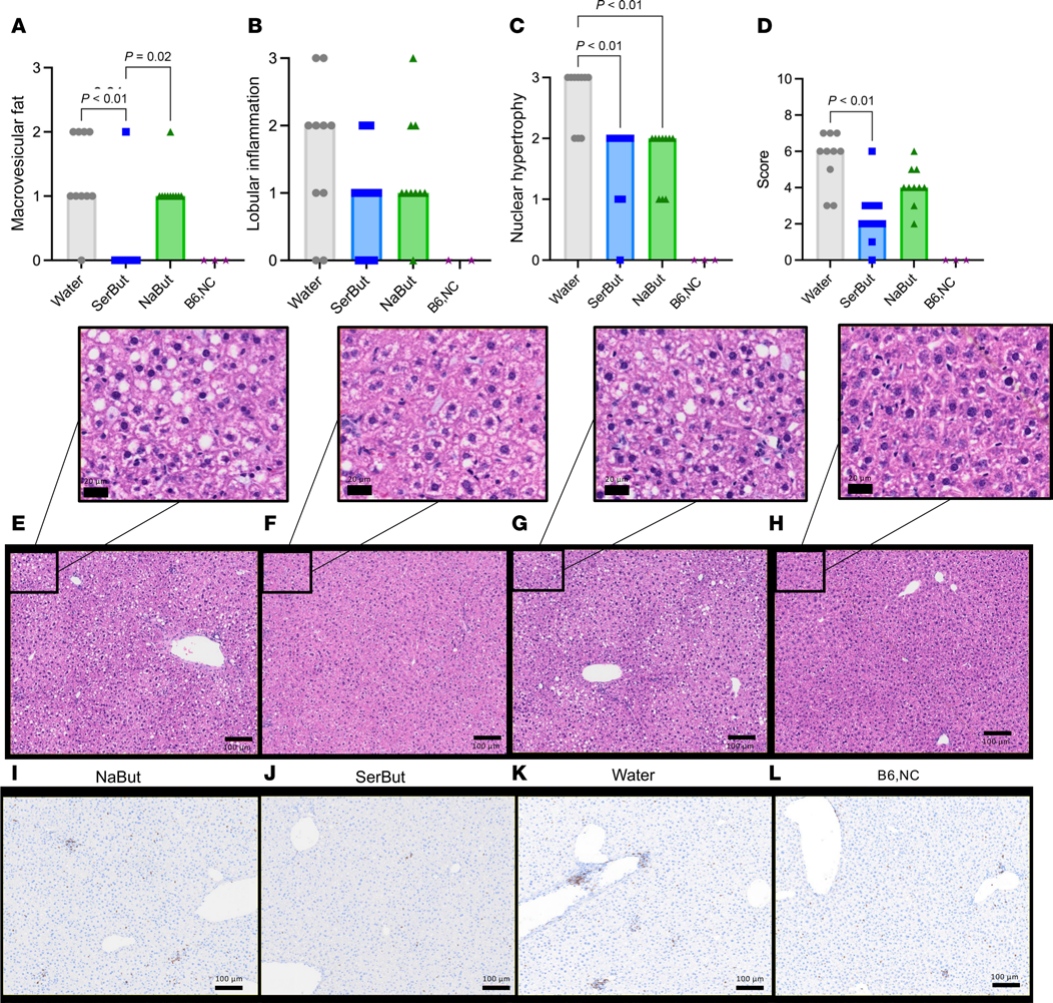
SerBut suppresses liver injury induced by HFD feeding. Histology of livers taken after 6 weeks of treatment. (**A**–**E**) Histological scoring of livers: (**A**) macrovesicular fat, (**B**) lobular inflammation, (**C**) nuclear hypertrophy, (**D**) overall score, and (**E**–**H**) representative images of H&E staining in (**E**) NaBut, (**F**) Serbut, (**G**) water, or (**H**) B6,NC. “B6,NC” denotes 3 age-matched C57BL/6 mice on a normal diet as a non-statistical comparison. Scale bars: 20 μm (top row) and 100 μm (bottom 2 rows). (**I**–**L**) Representative images of IHC-stained CD3^+^ immune cells in (**I**) NaBut, (**J**) SerBut, (**K**) water, (**L**) or age-matched WT controls. *n* = 10 per group in a single experiment for all groups. Kruskal-Wallis test for multiple comparisons was used. Score was calculated as the sum of scores displayed in **A**–**D** graded by a pathologist. **A** and **C** were graded using H&E-stained slides. **B** was graded using IHC-stained slides for CD3^+^ immune cells. Representative images were chosen based on average score for a particular treatment group.

It has previously been reported that butyrate can reduce fatty deposits, fibrosis, and inflammation in the liver (29, 45). Many patients with CVD have liver disease due to metabolic dysregulation, increased innate immune cell activation, and unresolved inflammation due to sustained NF-κB activation (46). Furthermore, many lipid-lowering drugs such as statins increase lipid uptake by the liver by increasing LDL-R expression, potentially inducing liver toxicity that prevents patients with liver disease from taking these medications (7). In the ApoE^–/–^ atherosclerotic model, HFD feeding induces hepatic lipid accumulation and inflammation in addition to plaque accumulation in the aorta (47). Thus, motivated by our finding of significantly reduced plasma ALT and AST in SerBut-treated groups (Figure 4, E and F), we further investigated SerBut’s potential beneficial effect on liver health under HFD feeding. Using a previously described liver histology scoring system, we investigated key features of nonalcoholic fatty liver disease and liver injury in mice: steatosis (macrovesicular fat), nuclear hypertrophy, and lobular inflammation of the liver (CD3^+^ immune cells) (48). Steatosis was significantly suppressed by SerBut treatment, but not by treatment with NaBut (Figure 5A). Neither SerBut nor NaBut significantly reduced CD3^+^ immune cell infiltration into the livers, indicating no change in lobular inflammation, which may be expected at such early time points in murine liver disease models (Figure 5B). Hepatocellular nuclear hypertrophy was suppressed by both SerBut and NaBut treatment (Figure 5C). Interestingly, both SerBut and NaBut treatment significantly suppressed LDL-R protein levels as shown by Western blot (Supplemental Figure 10). These data suggest that the reduction in circulating LDL-C levels via NaBut and SerBut treatment is not due to increased uptake of lipids via increased LDL-R expression. Thus, the overall summed histological score indicated reduced liver pathology in the ApoE^–/–^ HFD model due to SerBut, but not NaBut, treatment (Figure 5D). Representative images are shown (Figure 5, E–L).

We then asked whether SerBut’s effects on disease pathology and inflammation may be partially mediated by changes in lipid metabolism by measuring lipogenic and lipid oxidation gene expression by qPCR after treatment for 6 weeks (Supplemental Figure 10, F–L). SerBut-treated mice had significantly reduced expression of *Acly* compared with NaBut-treated mice but not compared to water-treated groups. No significant changes were seen in de novo lipogenesis, namely fatty acid synthase (*Fasn*) and acetyl CoA carboxylase 1 (*Acaca*). Carnitine palmitoyltransferase 1a (*Cpt1a*), the rate-limiting enzyme in β-oxidation, also was not significantly changed but showed a strong trend toward reduction compared with water-treated groups (*P* = 0.06). Taken together, these data suggest that reduction in liver lipogenesis nor increase in β-oxidation had measurable impact on phenotypic changes.

## Discussion

Butyrate, a key link between gut microbiota and metabolic health, has long been investigated for its beneficial effects in many metabolic and immune dysregulated states. The vast majority of either physiological (via microbial fermentation in the distal gut) or orally administered butyrate is metabolized by intestinal colonocytes as a primary energy source, limiting its antiinflammatory efficacy that might otherwise occur with systemic exposure. Since very little enters the circulation, the primary effect of orally dosed free butyrate is hypothesized to be on the intestinal lining and microbiome rather than distant organs and immune cells (49, 50). In the gut, butyrate promotes differentiation of colonic Treg cells via HDAC inhibition, allowing increased peripheral Treg cells by emigration (51, 52). Our lab previously engineered butyrate to escape the gut, utilizing esterification to a hydroxyl-containing amino acid, serine, hypothesized to allow butyrate transport into the portal blood through small intestinal amino acid transporters (28). This modification may additionally allow transit across the plasma membrane via transporters, dependent on the cell type. In this study, we assayed free, active butyrate in the gut contents and tissues separated by gut segment (Figure 2 and Supplemental Figure 2). We found that only in small intestinal tissue did SerBut-dosed mice show a significant increase in free, active butyrate by LC-MS/MS. These data, which we believe to be novel, support our hypothesis that SerBut escapes in the small intestine, evading consumption in the distal gut. Due to the promiscuous nature of amino acid transporters in the intestine and the failure of previous knockout experiments to inhibit amino acid transport, we did not investigate the specific amino acid transporter responsible for SerBut’s uptake (41), hypothesizing that SerBut transport is mediated by multiple amino acid transporters.

In this study, we investigated the progression of cardiometabolic disease in ApoE^–/–^ mice fed an HFD for 6 weeks in concurrence with SerBut or NaBut administered in drinking water ad libitum (Figure 3A). SerBut significantly suppressed atherosclerosis development, reducing plaque and necrotic core area in the aortic root and plasma LDL-C levels without affecting body weight (Figure 3H and Figure 4, A and H). SerBut did not induce liver injury or elevate LDL-R, as is common in some current standard-of-care therapeutics, but in fact inhibited liver injury induced by HFD feeding (Figure 4, E and F, Figure 5, and Supplemental Figure 10). These results support previous work showing butyrate’s protection against LPS-induced inflammatory liver injury through NF-κB suppression (21) and liver injury in an HFD-fed model (29) without suffering from dosing and translational limitations.

The in vivo studies described here have important limitations that may influence human translation. The ApoE^–/–^ murine model of atherosclerosis is advantageous due to its representation of inflammation yet has several limitations in recapitulation of human lipid metabolism. For example, since the lipid-lowering effect of statins is partially dependent on the presence of ApoE (53, 54), direct comparisons to the standard of care are precluded in this model. Furthermore, analysis of long-term suppression of liver injury would require longer periods of HFD feeding, as HFD-fed murine models of liver injury do not recapitulate the human disease until 8–16 weeks of HFD (55). The murine ApoE^–/–^ model lacks expression of the human lipoprotein gene CETP (encoding cholesteryl ester transfer protein), human CVD contributor lipoprotein(a), and differential bile acid composition. Thus, the lipid-lowering and immunometabolic modulatory properties of SerBut, especially those potentially due to modulation of liver metabolism, require further investigation to understand how these differences could impact translation.

Therapeutically targeting inflammation to reduce CVD has shown promise in recent trials such as the CANTOS (Cardiovascular Risk Reduction Study [Reduction in Recurrent Major CV Disease Events]) (14) and COLCOT (Colchicine Cardiovascular Outcomes Trial) (12) trials. In these trials, patients with relatively low levels of serum LDL-C or previous myocardial infarction events, respectively, saw reduced adverse cardiovascular events with immunotherapy. These results strongly suggest that inflammatory pathways play an important role in disease promotion. However, these trials used broad antiinflammatory medications: the IL-1β–neutralizing antibody canakinumab and the NLRP3 inflammasome inhibitor colchicine, respectively. A significant increase in lethal infections led to concerns about the safety of these drugs, although low doses of colchicine have been FDA approved. Despite these challenges, research on antiinflammatory approaches to treating and suppressing CVD became an important step toward better therapeutics (56).

Butyrate demonstrates antiinflammatory NF-κB–suppressing effects in myeloid cells by acting as an HDAC inhibitor (57), preventing NF-κB p65 from binding inflammatory gene promoters (58). Downstream of NF-κB activation, the NLRP3 inflammasome, in concert with caspase-1, cleaves pro-IL-1β to its active form. Active IL-1β stimulates enhanced secretion of IL-6 and IFN-γ along with other proinflammatory cytokines. We previously showed that SerBut maintains the HDAC-inhibiting activity of free butyrate (28). Here, we further demonstrate SerBut’s antiinflammatory effect, showing that SerBut suppresses NF-κB, downregulates iNOS expression, and reduces secretion of proinflammatory cytokines in macrophage-like cells in vitro (Figure 1, C–I).

We note that esterases that can cleave SerBut to yield bioactive butyrate are both extracellular and intracellular. Different relevant cell types may have many different import capabilities for both SerBut and hydrolyzed butyrate. SerBut presumably enables entry to the cytoplasm and liberation of free butyrate therein via intracellular esterases in vivo in various cell types. In addition to acting as a delivery vehicle for free butyrate, there are possible disparate molecular mechanisms of SerBut as a full construct that were not investigated in this study. There are a variety of tissue-specific esterases that could cleave SerBut, many of which are different between humans and mice (59). Increased bioavailability of free, active butyrate (Figure 2 and Supplemental Figure 2) shows that SerBut is indeed cleaved in assayed tissue types and leads to improvements in cardiometabolic health in each tissue of interest (aorta, heart, and liver). However, esterase expression differences between human and mice could lead to differing results in humans. It is yet to be seen whether these differences will prove beneficial or detrimental to the translation.

In murine atherosclerosis, SerBut reduced total immune cells, monocytes, and CCR2^+^ monocytes in the aorta compared with water-treated controls (Figure 3, M–O). This indicates suppression of myeloid activation, potentially resolving atherogenic inflammation and reducing recruitment (60). Systemically, SerBut reduced the proinflammatory cytokines IL-6 and IFN-γ in the plasma (Figure 3, Q and R) and circulating neutrophils (Figure 3S). A reduction in circulating monocytes, neutrophils, and platelets has previously been seen as a side effect in clinical trials investigating HDAC inhibitors (61). This side effect has inhibited the use of small molecule HDAC inhibitors for use in chronic diseases. It is an open question whether use of SerBut in humans would produce these complications; however, reduced inflammatory cells in circulation reduce the number of cells that can contribute to plaque formation, providing more benefit than complication. Importantly, we previously showed that although SerBut reduces innate immune activation, it does not dampen responses to vaccination in the presence of an adjuvant consisting of alum admixed with the TLR4 agonist monophosphoryl lipid A (28). Questions remain on the effect of SerBut treatment on innate immune responses to infection; however, previous reports suggest that the antiinflammatory effects of butyrate are context dependent (28).

In summation, we have demonstrated that systemically bioavailable butyrate dosed through SerBut is able to modulate immunity, thus suppressing atherosclerosis progression and liver pathology in the ApoE^–/–^ HFD murine model of atherosclerosis. Butyrate has long been of interest in cardiometabolic disease, but its exploration has been limited by its low bioavailability and aversive sensory experience. Our results show that addressing these challenges significantly accentuates the benefit of butyrate dosing in the ApoE^–/–^ model of atherosclerosis compared with treatment with NaBut. SerBut’s liver protective effect was also accentuated compared with NaBut, likely attributable to enhanced exposure of the liver as indicated in our biodistribution study. This information may be useful in considering simple, low-toxicity combinations to reduced progression of cardiometabolic disease, including in patients with previous liver disease, liver injury, or mutations in the ApoE gene diminishing their response to statins (5, 53, 54).

## Methods

Further information can be found in Supplemental Methods.

### Sex as a biological variable.

This study was conducted using female ApoE^–/–^ mice. Sex differences are known to influence the development of atherosclerosis and metabolic disease (62). For example, female mice typically develop larger aortic root lesions earlier than male mice. Female mice also exhibit a stronger adaptive immune response to oxidized LDL and a more robust innate immune response to TLR stimulation (63, 64). In contrast, pharmacological interventions such as PPARγ agonists and IFN-γ deficiency have shown therapeutic benefits primarily in male mice (65, 66). These observations suggest that sex may influence the magnitude of therapeutic response in ApoE^–/–^ mice. However, because inflammation is a central feature of atherosclerosis in both sexes, and because of the complex immunological mechanisms involved in our study, it is difficult to predict whether sex-specific differences would enhance or attenuate the observed effects. To reduce biological variability and maintain clarity in data interpretation, we conducted all experiments in a single sex.

### Synthesis of SerBut.

SerBut was synthesized as described previously (28). Briefly, L-serine (20 g, 0.19 mol) was added to trifluoroacetic acid (200 mL), and the suspension was stirred for 30 minutes until everything dissolved. Butyryl chloride (25.7 mL, 0.23 mol) was then added to the solution, and the mixture was stirred for 2 hours at room temperature. The reaction was then transferred to an ice bath, and diethyl ether (500 mL) was added, which resulted in precipitation of a white solid. The resulting fine white precipitate was collected by filtration, washed with cold diethyl ether, and dried under vacuum to yield 26.3 g of *O*-butyryl-L-serine (0.15 mol, 79%). The final product was confirmed by ^1^H NMR (500 MHz, DMSO-d_6_) [ppm]: 0.88 (3H, t), 1.55 (2H, m), 2.32 (2H, t), 4.30 (1H, t), 4.43 (2H, d), 8.66 (2H, s), 14.06 (1H, s).

### Derivatization and LC-MS/MS detection of butyrate.

Samples were prepared and derivatized as described previously (28). Stock solutions were prepared in water/acetonitrile (ACN) (1:1 v/v): 3-nitrophenylhydrazine (NPH), and 1-ethyl-3-(3-dimethylaminopropyl)carbodiimide (EDC) (in 1% pyridine). The internal standard, 4-methylvaleric acid, was added. Samples were mixed with NPH and EDC stocks at a 1:1:1 volume ratio. The mixture was heated in a heating block at 60°C for 30 minutes. Samples were filtered through 0.22 μm filters and stored at 4°C before analysis.

An Agilent 6460 Triple Quad MS-MS was used to detect the derivatized butyrate. Both derivatized butyrate-NPH and 4-methylvaleric-NPH were detected in negative mode. Column: Thermo Fisher Scientific C18 4.6 × 50 mm, 1.8 μm particle size, at room temperature. Mobile phase A: water with 0.1% v/v formic acid. Mobile phase B: ACN with 0.1% v/v formic acid. Injection volume: 5.0 μL. Flow rate: 0.5 mL/min. Gradient of solvent: 15% mobile phase B at 0.0 minutes; 100% mobile phase B at 3.5 minutes; 100% mobile phase B at 6.0 minutes; 15% mobile phase B at 6.5 minutes. The MS conditions were optimized using pure butyrate-NPH or 4-methylvaleric-NPH at 1 mM. The fragment voltage was set to 135 V, and the collision energy was 18 V. Multiple reaction monitoring (MRM) of 222 → 137 was assigned to butyrate, and MRM of 250 → 137 was assigned to 4-methylvaleric acid as the internal standard. The ratio between MRM of butyrate and 4-methylvaleric acid was used to quantify butyrate concentration.

### LPS-induced activation of RAW 264.7 macrophages and RAW-blue macrophages.

RAW 264.7 (ATCC, TIB-71) macrophages were cultured at 37°C and 5% CO_2_ in DMEM (Gibco) supplemented with 1 mM HEPES, 1 mM sodium pyruvate, 10% FBS, and penicillin/streptomycin. Cells were plated in round-bottom 96-well plates at 100,000 cells per well and incubated with different concentrations of either NaBut or SerBut (0.15–12 mM) for 24 hours. Cells were then rinsed with PBS and then stimulated with 200 ng/mL LPS in media for 6 hours. The cell culture supernatant was collected and analyzed by LEGENDPlex to analyze the concentrations of cytokines (BioLegend). Cell phenotype was analyzed using flow cytometry (Cytek Aurora).

NF-κB inhibition was assayed using the NF-κB-SEAP reporter cell line, RAW-Blue (InvivoGen, raw-sp). Cells were plated in round-bottom 96-well plates at 100,000 cells per well and cocultured with different concentrations of either NaBut or SerBut (0.15–12 mM) for 24 hours. After the addition of butyrate, cells were rinsed with phosphate buffered saline (PBS) and then stimulated with 200 ng/mL LPS in media for 6 hours.

### Cell viability assay.

CellTiter 96 AQueous one Solution Cell Proliferation Assay (MTS) (Promega, G3582) was used according to the manufacturer’s instructions to analyze cytotoxicity of SerBut and NaBut at various concentrations (0.15–12 mM) in RAW 264.7 macrophages. Viability values were normalized to untreated cells (100%) and Triton X-100–treated cells (0%). Since absorbance is directly proportional to the number of living cells in culture, values greater than 100% indicate cell growth compared with controls.

### Mice.

Female ApoE^–/–^ mice, aged 6–8 weeks, were purchased from The Jackson Laboratory (stock 002052; B6.129P2-*Apoe^tm1Unc^*/J). Female C57BL/6 mice aged 6–8 weeks were purchased from Charles River (strain code 027, Charles River). C57BL/6 and ApoE^–/–^ mice were maintained in a specific pathogen–free facility at the University of Chicago. Mice were maintained on a 12-hour light/dark cycle at a room temperature of 20°C–24°C.

### Biodistribution of SerBut.

ApoE^–/–^ mice on 6 weeks of HFD were orally gavaged with 272 mM butyrate, equal to approximately 24 mg butyrate (52 mg SerBut or 30 mg NaBut) or PBS. Three hours after administration, mice were anesthetized under isoflurane, and blood was collected via submandibular vein into EDTA-coated tubes. The tubes were centrifuged, and plasma was collected and frozen at –80°C. Mice were then pressure perfused with 30 mL of PBS containing 1 mM EDTA via the left ventricle after severing the inferior vena cava. Organs, including liver, MLNs, ILNs, spleen, lung, heart, aorta, small intestine, and large intestine were collected and then transferred to –80°C until further processing. To extract butyrate from plasma or organs, a 1:1 (v/v) ACN/water solution was used. Plasma was mixed 1:1 with the ACN/water solution and centrifuged to remove denatured proteins. Organs were weighed, transferred to Lysing Matrix D tubes (MP Biomedicals), and combined with the 1:1 v/v ACN/water solution. Samples were then lysed using a FastPrep-24 5G homogenizer (MP Biomedicals) and centrifuged. The supernatants were collected for butyrate measurement by LC-MS/MS. The final butyrate content in each organ was normalized by organ weight.

### Drinking water SerBut administration in ApoE^–/–^ model.

For 6 weeks, mice were freely administered water, water containing 150 mM SerBut, or water containing an equimolar amount of NaBut. SerBut water was pH adjusted using 10 M NaOH to a final pH of 7.4. NaBut water was not pH adjusted and had a pH of approximately 8. In concert, mice were fed an HFD with 42% from fat and 0.2% total cholesterol (Envigo, TD-88137) ad libitum. After 6 weeks, mice were euthanized. The lMLNs, ILNs, spleen, heart, and aorta were harvested and processed for flow cytometry analysis (67). The liver was harvested and part was homogenized in tissue protein extraction reagent buffer (Invitrogen, 78510) supplemented with protease inhibitor mini tablets (Thermo Fisher Scientific, PIA32955) for Western blotting and qPCR. The remaining liver was fixed with 4% paraformaldehyde and embedded in paraffin for histological analysis.

### Flow cytometry and antibodies.

Flow cytometry was performed using a BD LSRFortessa or a Cytek Aurora full-spectrum cytometer. Data were analyzed using FlowJo version 10.8.0. eFluor 780 Viability Dye (Invitrogen, 65-0865-14) and antibodies against the following markers were used in the RAW 264.7 macrophage in vitro studies: CD86 (BV421; BD Horizon, catalog 564198), CD80 (BC650; BioLegend, clone 16-10A1, catalog 104732), and iNOS (APC; Invitrogen, clone CXNFT, catalog 17-5920-9). Live/Dead Blue (Invitrogen, 123105A) and antibodies against the following markers were used in the atherosclerosis mouse models: F4/80 (FITC; BioLegend, clone BM8, catalog 123108), CD3e (BV605; BD Biosciences, clone 145-2C11, catalog 563565), CD4 (BUV496; BD Biosciences, clone GK 1.5, catalog 612952), RORγt (AF647; BD Biosciences, clone Q31-378, catalog 562682), FoxP3 (AF488, BD Biosciences, clone MF23, catalog 560403), CCR2 (PE; R&D Systems, catalog FAB5538P), Ly6C (APC-Cy7; BioLegend, catalog 128026, clone HK1.4), Ly6G (AF488; BioLegend, catalog 127626), CD8 (APC-Cy7; BioLegend, clone 53-6.7, catalog 100714), CD45 (BUV805; Invitrogen, clone 30-F11, catalog 368-0451-82), and CD25 (PerCP-Cy5.5; BioLegend, clone PC61, catalog 102030).

### Flow cytometry of the aorta.

As previously described (67), mice were pressure perfused with 30 mL of PBS via the left ventricle after severing the inferior vena cava. The heart with the ascending and descending aorta to the diaphragm was harvested. The perivascular adipose tissue was carefully removed by microdissection while keeping the adventitia intact. The aorta was then cut at the base of the heart, placed in digestion media, and cut into 5 mm long pieces. To create a single cell suspension, digestion media consisted of DMEM supplemented with 0.5 mM CaCl_2_, 2.5 mM MgCl_2_, 125 U/mL collagenase XI (Sigma-Aldrich), 450 U/mL collagenase I (Sigma-Aldrich), 60 U/mL hyaluronidase, (Sigma-Aldrich), and 60 U/mL DNase I (Sigma-Aldrich). After 45 minutes of digestion at 37°C with orbital shaking, aortas were passed through a 70 μm cell strainer, rinsed, pelleted, and plated into a U-bottom 96-well microplate for staining. Cells were washed in PBS and stained for 15 minutes on ice with 1:500 Live/Dead Fixable Violet Dye (Invitrogen) and 1:200 anti-mouse CD16/CD32 (clone 93, BioLegend). Cells were washed in PBS supplemented with 2% FBS (FACS buffer) and stained for 30 minutes on ice with surface antibodies at a 1:200 dilution in a 1:1 mixture of FACS buffer and Brilliant Stain Buffer (BD Biosciences). Cells were washed with FACS buffer and PBS and fixed with 2% paraformaldehyde on ice for 20 minutes. Cells were washed twice with PBS and resuspended in FACS buffer. Precision count beads (BioLegend) were used to determine absolute cell counts. In this study, antibodies used included those against CD16/CD32 (BioLegend, clone 93, catalog 101302), CCR2 (PE; R&D Systems, catalog FAB5538P), CD11b (BV605; BioLegend, clone M1/70, catalog 101257), CD11c (PE-Cy7; BD Biosciences, clone HL3, catalog 558079, BD Biosciences), CD19 (APC; BioLegend, clone 1D3, catalog 152412), CD3e (BUV395; clone 145-2C11, catalog 563565), CD45 (BV786; BD Biosciences, clone 30-F11, catalog 564225), F4/80 (BUV496; BD Biosciences, clone T45-2342, catalog 750644), I-A/I-E (PerCP-Cy5.5; BioLegend, clone M5/114.15.2, catalog 107626), Ly-6C (APC-Cy7; BioLegend, clone HK1.4, catalog 128026), and Ly-6G (FITC; eBioscience, clone 1AB, catalog 11-9668-80).

### Sectioning and Oil Red O staining of aortic root.

Hearts were fixed in 4% paraformaldehyde for 24 hours and cryopreserved with sucrose (15% sucrose for 24 hours, 30% sucrose for 24 hours, 1:1 30% sucrose and OCT) before being embedded in OCT (Thermo Fisher Scientific). Frozen embedded samples were sectioned at 8 μm until all 3 leaflets were visible. Aortic root sections were brought to room temperature and rehydrated with PBS. Staining solution was freshly prepared by mixing 3:2 Oil Red O (0.5% in isopropanol, Sigma Aldrich) to water. The solution was filtered through a 0.22 μm PES syringe filter (Millipore) and pipetted onto sections. After 15 minutes in a sealed dark chamber, sections were washed 3 times with water and imaged. Images were analyzed using QuPath (v0.5.0) (https://qupath.github.io). Each leaflet was circled broadly to include the plaque and then subsequently trained on the QuPath pixel classifier using the software’s artificial neural network. Training images were then tested and validated to identify the plaques to calculate their area and exclude non-plaque structures in the image. Plaque sizes were subsequently averaged and used to determine percentage Oil Red O–positive plaque area in the leaflet.

### Immunohistochemistry and Stary plaque severity grading.

Frozen aortic root sections were fixed in Zamboni fixative for 10 minutes. After TBS wash, slides were loaded on Leica Bond RX automated stainer. Anti–mouse CD68 antibody (BioLegend, catalog 137001, clone FA-11; 1:4000) was applied to tissue sections for 60 minutes of incubation at room temperature. Following TBS wash, tissue sections were incubated with rabbit anti-rat IgG (Thermo Fisher Scientific, 31219) 30 minutes. The antigen-antibody binding was detected using Polymer-HRP (Leica Biosystems, Bond Polymer Refine Detection kit, DS9800). The slides were coverglassed by autocoverslipper (Tissue-Tek Glas g2). Stary plaque severity grading was done as previously described (42). Briefly, each individual mouse was scored if there were at least 2 sections showing all 3 leaflets of the aortic valve that could reasonably be scored. Each leaflet was scored individually per aortic valve and subsequently averaged per section. All sections were averaged per mouse for a final score. Sections were graded blinded. Table 1 explains the scoring scale. Quantitative analysis of plaque features was performed using a classifier in QuPath (v.05.0).

### Immunofluorescent staining of the aortic root.

Aortic root sections were brought to room temperature and rehydrated with PBS and permeabilized with 10% dimethyl sulfoxide (DMSO). Sections were blocked with 0.05% casein in PBS and stained overnight at 4°C with rat monoclonal anti–mouse CD68 at 1:400 (BioLegend, clone FA-11) and rabbit polyclonal anti–mouse iNOS at 1:50 (Proteintech, 18985-1-AP). Sections were then washed with PBS with 0.05% Tween 20 and incubated for 1 hour at room temperature with secondary antibodies Alexa Fluor 488 donkey polyclonal anti-rabbit IgG at 1:500 (Invitrogen, A21208) and Alexa Fluor 555 donkey polyclonal anti-rabbit IgG at 1:500 (Invitrogen, A31572). Sections were washed again in PBS with 0.1% Tween 20 and then PBS and mounted with ProLong Gold Antifade Mountant with DAPI (Invitrogen). Slides were imaged with a DMI8 inverted fluorescence microscope (Leica Microsystems) and analyzed with QuPath (v0.5.0).

### Blood chemistry and inflammatory cytokines in plasma.

Blood chemical analysis was performed on an Alfa Wasserman ACE Axcel Chemistry Analyzer. Reagent kits from Alfa Wasserman were used according to manufacturer’s instructions to analyze plasma: lipase (catalog SA1045), HDL-C (catalog SA2038, LDL-C (catalog SA1040), AST (catalog SA1053), ALT (catalog SA1052). A LEGENDPlex was utilized to analyze the concentrations of inflammatory cytokines (BioLegend).

### Histological grading and scoring of livers.

Sections (8 μm thick) were stained. Digital image files were created with an Olympus VS200 Research Slide Scanner (Olympus/Evident) with a Hamamatsu ORca-Fusion camera (Hamamatsu Photonics) at ×40 magnification. Individual images were created with the OlyVIA Viewer software (Olympus/Evident). Scoring and grading was performed by a pathologist as previously described (48). Immunohistochemistry grading was informed by age-matched WT controls. In age-matched WT controls, CD3^+^ immune cells were seen scattered without distinct aggregate formation nor associated tissue reaction. In an ×400 field, we observed up to 25 CD3^+^ immune cells scattered in an even distribution, with rare events of up to 10 periductal CD3^+^ immune cells but with no associated ductal or parenchymal reaction. A score of 1 was given to samples with one focus in which more than 10 periductal CD3^+^ lymphocytes are found in an ×200 field, suggesting mild periductal inflammation. Furthermore, a score of 2 corresponds to 2 to 5 instances of more than 10 periductal CD3^+^ immune cells in an ×200 field. Any sample exceeding this threshold was scored a 3.

### Statistics.

Statistical analysis and plotting of data were performed using Prism 10.0.2 (GraphPad), as indicated in figure legends. One-way ANOVA with Tukey’s, Brown-Forsythe, and Welch’s (if standard deviations [SDs] were significantly different), or Kruskal-Wallis post hoc test (if data were not normally distributed) were used for multiple comparisons. Bartlett’s test and Brown-Forsyth test were performed to determine whether assumption of equal SDs was appropriate. If *P* was less than 0.05, Brown-Forsythe and Welch’s ANOVA tests were performed assuming different SDs. Normality analysis was done by Shapiro-Wilk test. An unpaired Student’s *t* test was used for pairwise comparisons. Welch’s *t* test was used if SDs were significantly different. If data were not normally distributed, determined by Shapiro-Wilk normality test, a Mann-Whitney test was used.

### Study approval.

All protocols used in this study were approved by the Institutional Animal Care and Use Committee of the University of Chicago.

### Data availability.

All raw data can be found in the Supporting Data Values file. Code and 16S rRNA sequencing data are available at GitHub: https://github.com/tarynbeckman/SerBut

## Author contributions

JAH oversaw all research. TNB and LRV designed all experiments. TNB synthesized materials. TNB, LRV, AJS, EB, GB, SNDM, JWR, SG, LZ, AT, and KCR performed experiments. TNB, LRV, SNDM, JWK, AW, AT, and AJS analyzed experiments. SC assisted in analysis of experiments. OD, JWR, ALL, and EBC provided bioinformatics support. TNB, LRV, and JAH wrote the manuscript. All authors contributed to writing the manuscript and approved the submitted version.

## Supplementary Material

Supplemental data

Unedited blot and gel images

Supporting data values

## Figures and Tables

**Table 1:**
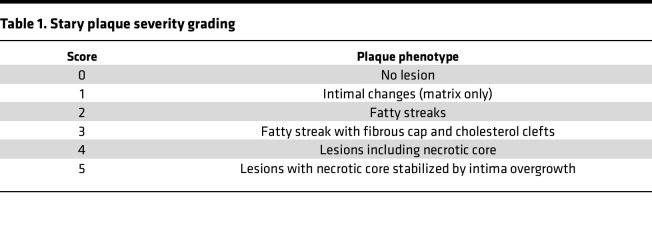
Stary plaque severity grading

## Version 1 08/08/2025

Electronic publication
