# Supplementary material for: A prometabolite strategy inhibits cardiometabolic disease in an ApoE–/– murine model of atherosclerosis
Source: JCI Insight. 2025 Aug 8;10(15):e191090. doi: 10.1172/jci.insight.191090 (PMC12333940; doi:10.1172/jci.insight.191090)
Supplement: Supplemental data [file jciinsight-10-191090-s236.pdf]

Supplemental Methods

16S rRNA Sequencing

Feces were collected and stored at -80°C. Sequencing was performed as previously described[39]. Bacterial DNA was extracted using the QIAamp PowerFecal Pro DNA kit (Qiagen). The V4-V5 hypervariable region of the 16S rRNA gene from the purified DNA was amplified. Illumina sequencing-compatible unique dual index adapters were ligated onto pools using the QIAsep 1-step amplicon library kit (Qiagen). Library quality control was performed using Qubit and Tapestation before sequencing on an Illumina MiSeq platform at the Duchossois Family Institute Microbiome Metagenomics Facility at the University of Chicago. This platform generates forward and reverse reads of 250 bp which were analyzed for amplicon sequence variants (ASVs) using the divisive amplicon denoising algorithm dada2 (v1.18.0) as our default pipeline for processing MiSeq 16S rRNA reads with minor modifications in R (v4.0.3). Specifically, reads were first trimmed at 190 bp for both forward and reverse reads to remove low quality nucleotides. Chimeras were detected and removed using the default consensus method in the dada2 pipeline. Then, ASVs with length between 320 bp and 365 bp were kept and deemed as high quality ASVs. Taxonomy of the resultant ASVs were assigned to the genus level using the RDP classifier (v2.13) with a minimum bootstrap confidence score of 80. The ASV tables, taxonomic classification and sample metadata were compiled using the phyloseq data structure[64]. Subsequent 16S rRNA relative abundance analyses and visualizations were performed using R version 4.2.2 (R Development Core Team).

Microbiota Community Variation

All statistical analyses and graphical representations were performed in R using the packages Vegan (v2.6-4), Phyloseq (v1.42.0), Caret (v6.0-94), ggplot2 (v3.4.4), Ape (v5.7-1), and Corrplot (v0.92).

Taxa were agglomerated at genus level using the tax_glom function (Phyloseq package) in R. Reads were rarefied to an even depth using the rarefy_even_depth function (Phyloseq package). Finally, abundances were relativized using the transform_sample_counts function (Phyloseq package). To further clean data, null values and predictors with near-zero variances were removed using the nearZeroVar function (Caret package). To minimize the influence of cage effects, taxa that were present only in less than 10% of samples were removed.

Phylogenic trees were created using the rtree function (Ape package), which was subsequently merged onto the phyloseq object. Beta-diversity was calculated via weighted and unweighted Unifrac distances, as well as Bray-Curtis distances. The Ordinate function (Phyloseq package) was used with PCoA method to calculate distances for both weighted and unweighted Unifrac distances. Permutational multivariate analyses of variance using distance matrices were calculated using the adonis2 function (Vegan package). Plotting of Beta-diversity was performed using the plot_ordination function (Phyloseq Package, ggplot2 package).

Association Between Atherosclerosis Pathology and Treatment Variables (Exogenous Butyrate, Seryl Modification of Exogenous Butyrate, and Microbiome Variables)

Linear modelling was performed in a manner similar to Valles-Collomer et al[65]. Associations between taxa, butyrate, or Seryl modification of butyrate and pathology readouts after partialling out the effects of the other two treatment variables as covariates were assessed by fitting linear models. Pathology readouts (eg. Plaque % in the aortic root, LDL-C/Chol) were used as

responsive variables, and treatment variables as explanatory/predictive variables. Multivariate linear models were performed using the lm R function (stats package). Standardization of predictor and response variables was performed using the scale function in base R. Associations were included as significant at P<0.025. In Supplemental Figure 5, values are shown as Rho correlation coefficients transformed from β coefficients as calculated below.

Null Linear Model:

$$Y_{pathology} = 0 + \beta_0 + z_{seryl}\beta_{Seryl} + x_{Gi}\beta_{Gi} + Error$$

Alternative Linear Model:

$$Y_{pathology} = x_{butyrate}\beta_{Butyrate} + \beta_0 + z_{seryl}\beta_{Seryl} + x_{Gi}\beta_{Gi} + Error$$

$$\beta_{Butyrate} = Rho(\frac{S_{pathology}}{S_{Butyrate}})$$

$$Rho = \beta_{Butyrate} * (\frac{S_{Butyrate}}{S_{Pathology}})$$

qPCR analysis of the Liver

Frozen mouse liver was powderized and added to 1 mL of TRIzol (Invitrogen, 15596018). Samples were homogenized at 4°C using a FastPrep-24 Homogenizer (MP Biomedicals) for 3 cycles of 20 seconds at 4.0 m/s, using 2 mL FastPrep tubes and caps (MP Biomedicals, 5076400 and 5064002) with 6 beads of Lysing Matrix S (MP Biomediacals, 6925000). Samples were rested for 1 minute in between cycles. After homogenization, RNA was extracted according to the TRIzol user guide and resuspended in RNase-free water. RNA concentrations were measured using a NanoDrop One^{C}.

To make cDNA, 2 ug of RNA were reverse-transcribed using the High Capacity RNA to cDNA Kit (Applied Biosystems, 4387406) according to the user guide. To determine gene expression, we performed quantitative real-time qPCR using TaqMan Fast Advanced Master Mix (Applied Biosystems, 4444557) and gene-specific probes (Thermo Fisher Scientific, Acaca: Mm01304277_m1 and Acly Mm00652520_m1). qPCR was performed using a QuantStudio 3

Real-Time PCR System (Applied Biosystems). Samples were assayed in triplicate and normalized to their respective endogenous control (Rps12: Mm03030276_g1). Gene expression levels were quantified using the $\Delta\Delta C_T$ method and relative expression of each sample was normalized to a water treated sample.

Supplemental Figures

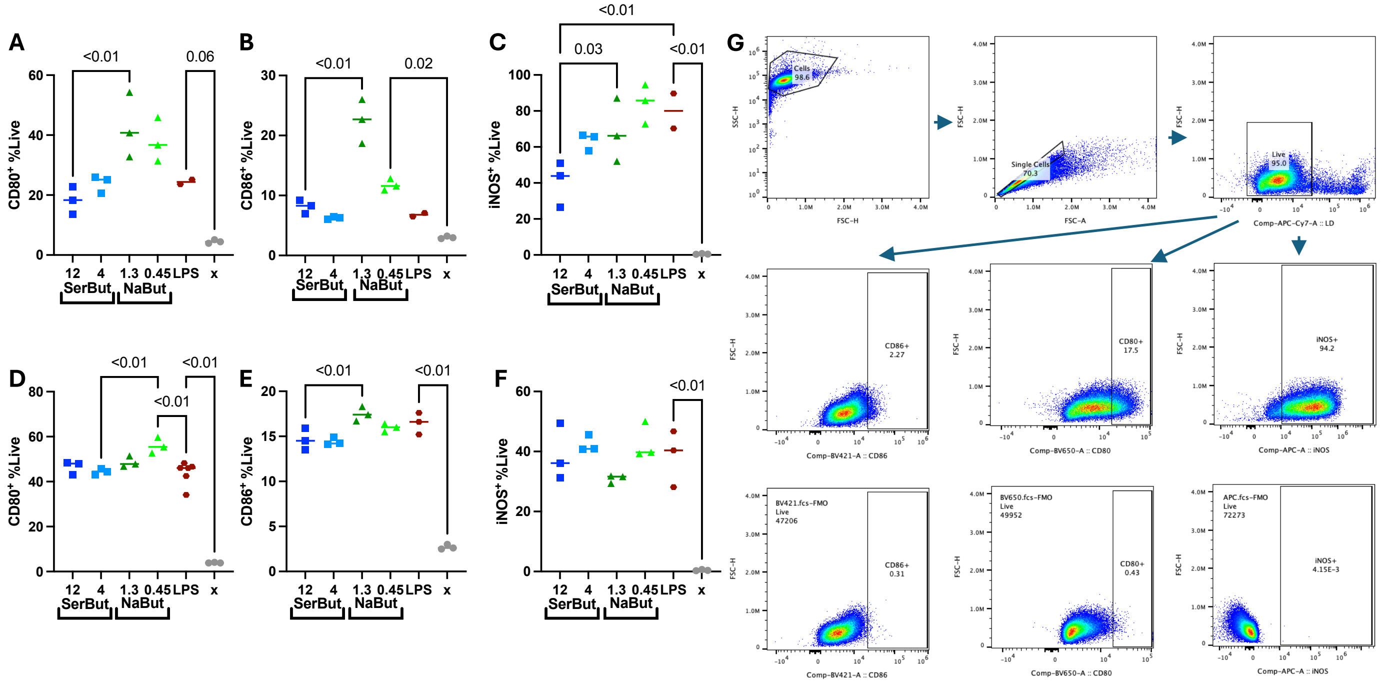

Supplemental Figure 1. Flow Cytometry of RAW 264.7 Macrophages A-C. Pretreatment of butyrate followed by LPS stimulation for 12 hr in RAW 264.7 Macrophages. **D-E.** LPS stimulation for 12 hr followed by butyrate treatment. **G.** Gating strategy. Statistical analyses were performed using a one-way ANOVA with a Tukey's, Welch's (if standard deviations were significantly different by Bartlett and Brown-Forsyth tests), or Kruskal Wallis's (if data were not normally distributed determined by Shapiro-Wilk test) post-test. P-values less than 0.10 are shown.

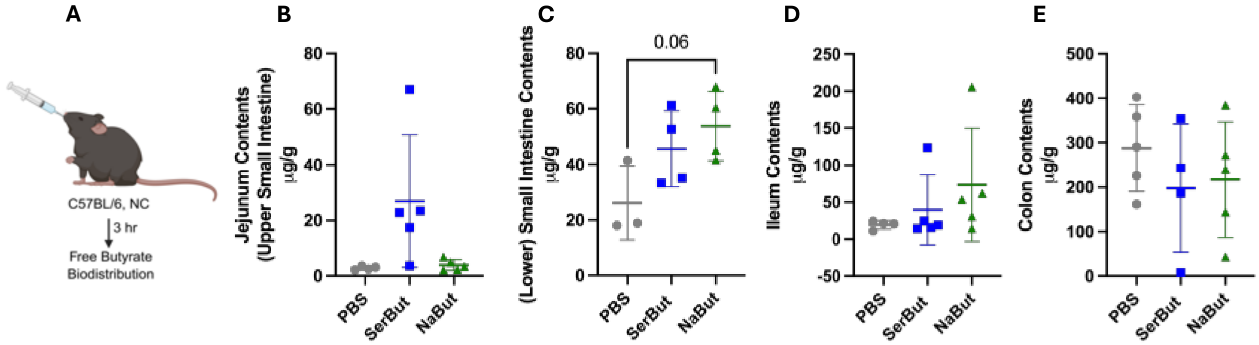

Supplemental Figure 2. Seryl modification increases oral bioavailability of butyrate in atherosclerotic organs. A. Experimental schema of C57BL/6 mice on a NC diet who received a single equimolar gavage of 52 mg SerBut or 30 mg NaBut. After 3 hr, mice were perfused, and the amount of butyrate was measured in segmented intestinal contents separated from the intestinal tissue including the **B.** jejunum contents, **C.** lower small intestine contents, **D.** Ileum contents, and **E.** colon contents. Quantification was performed by LCMS/MS upon derivatization with 3-nitrophenylhydrazine. N=5 mice per group. Free butyrate (μg) was normalized to tissue weight (g). Data points represent individual mice displayed with median + s.e.m. Statistical analyses were performed using a one-way ANOVA with a Tukey's, Welch's (if standard deviations were significantly different by Bartlett and Brown-Forsyth tests), or Kruskal Wallis's (if data were not normally distributed determined by Shapiro-Wilk test) post-test. P-values less than 0.10 are shown.

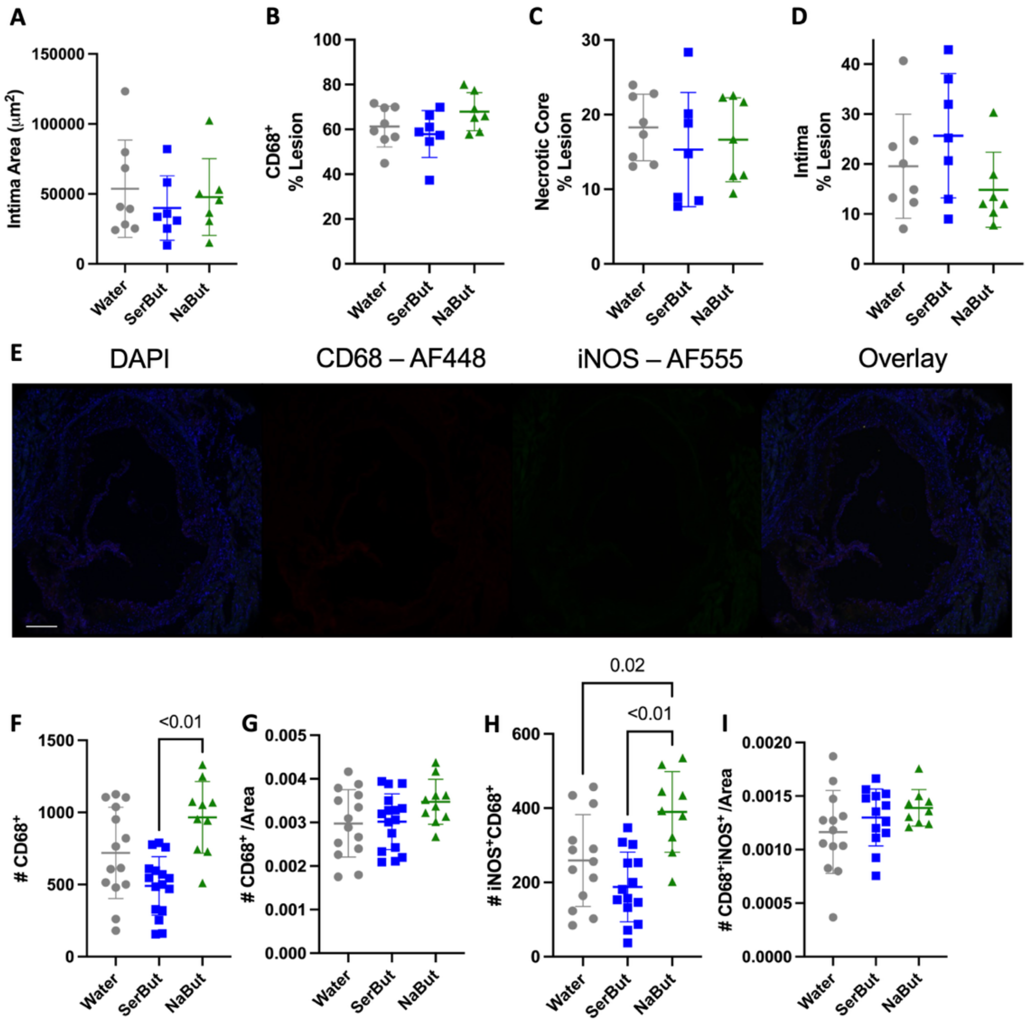

Supplemental Figure 3. Immunofluorescence and CD68^{+} IHC analysis of macrophages in the aortic root. A-D. Analysis of CD68^{+} Immunohistochemistry-stained aortic roots collected at 6 weeks of HFD and treatment. **A.** Quantified intima of CD68^{+} IHC stained plaque. **B.** CD68^{+} area normalized to total atherosclerotic lesion. **C.** Necrotic Core area normalized to total atherosclerotic lesion. **D.** Intima normalized to total atherosclerotic lesion. **E.** Immunofluorescence staining Isotype Controls (from left to right): DAPI Isotype control ranging from 101.9-4497, CD68 Isotype control ranging from 102.6-15648.4, iNOS Isotype control ranging from 72.6-28494 and Overlay. Positive cells were identified based on mean fluorescent intensity thresholding. **F.** Quantification of CD68^{+} macrophages, **G.** CD68^{+} macrophages normalized to plaque area, **H.** iNOS^{+} CD68^{+} inflammatory macrophages, and **I.** iNOS^{+}CD68^{+} inflammatory macrophages normalized to plaque area. Statistical analyses were performed using a one-way ANOVA with a Tukey's, Welch's (if standard deviations were significantly different by Bartlett and Brown-Forsyth tests), or Kruskal Wallis's (if data were not normally distributed determined by Shapiro-Wilk test) post-test.

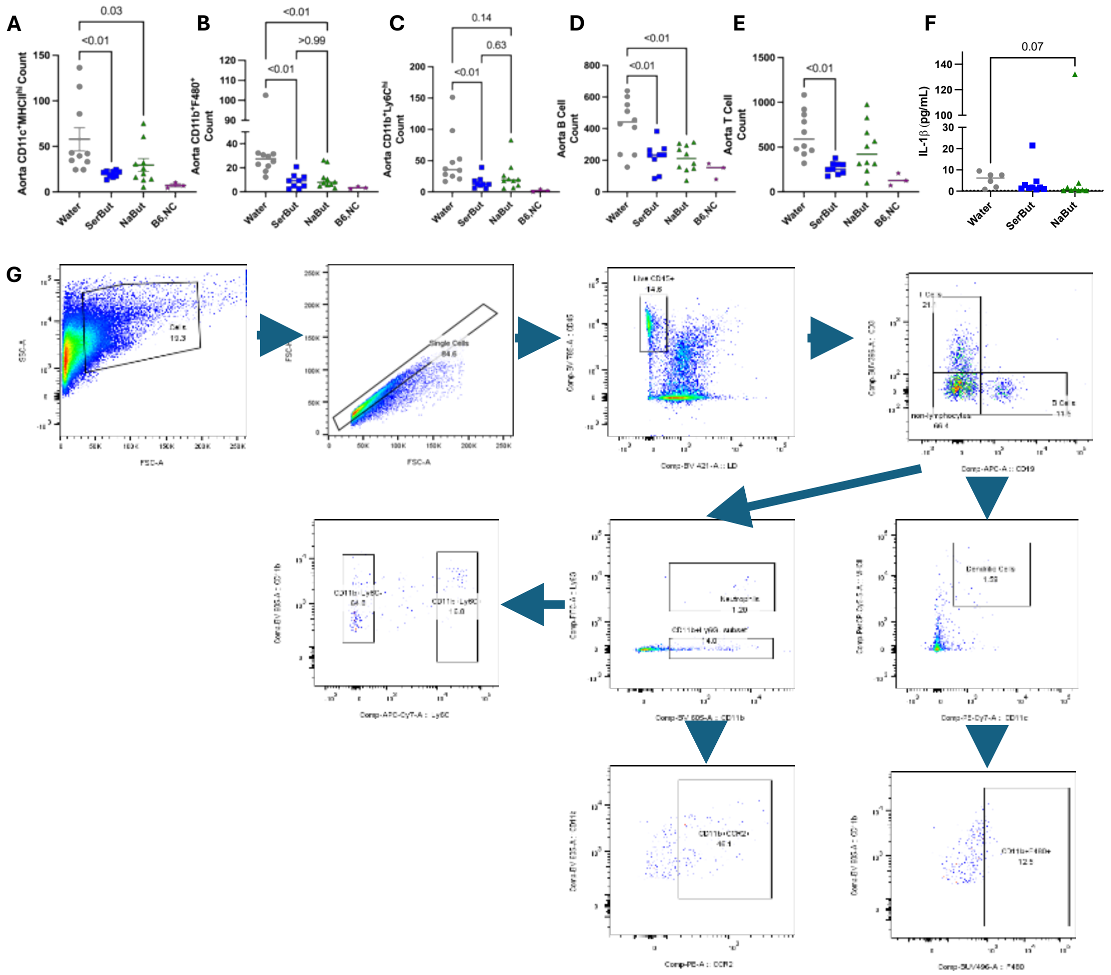

Supplemental Figure 4. A-E. Aorta flow cytometry: **A.** $CD11c^{+}MHCII^{hi}$ dendritic cells, **B.** Macrophages, **C.** $CD11b^{+}$ $Ly6C^{hi}$ myeloid cells, **D.** $CD19^{+}$ B cell count, and **E.** $CD3^{+}$ T cell count. **F.** Plasma IL-1β. **G.** Aorta flow cytometry gating strategy.

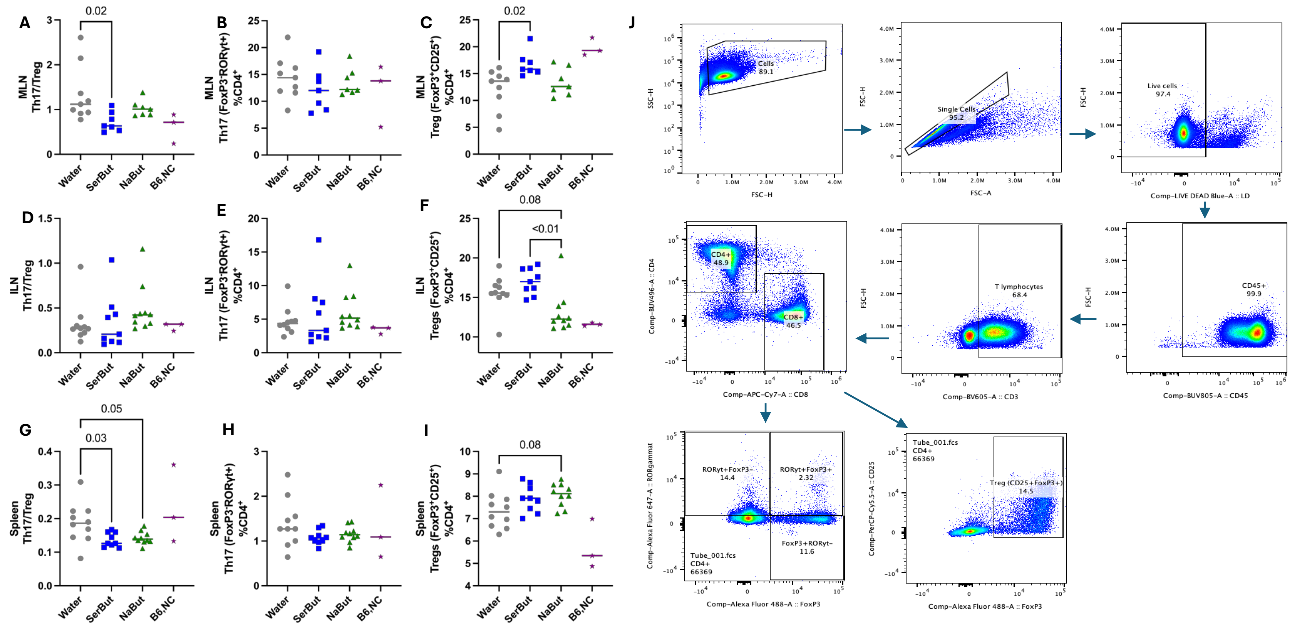

Supplemental Figure 5. Flow cytometry of ratio of Th17 T cells to Treg T cells, proportion of CD4+ T cells that are Th17 cells, and proportion of CD4+ T cells that are Treg T cells. A-C. Mesenteric lymph node flow cytometry. **D-F** Inguinal Lymph node flow cytometry. **G-I.** Spleen flow cytometry. **J.** Gating strategy. Statistical analyses were performed using a one-way ANOVA with a Tukey's, Welch's (if standard deviations were significantly different by Bartlett and Brown-Forsyth tests), or Kruskal Wallis's (if data were not normally distributed determined by Shapiro-Wilk test) post-test.

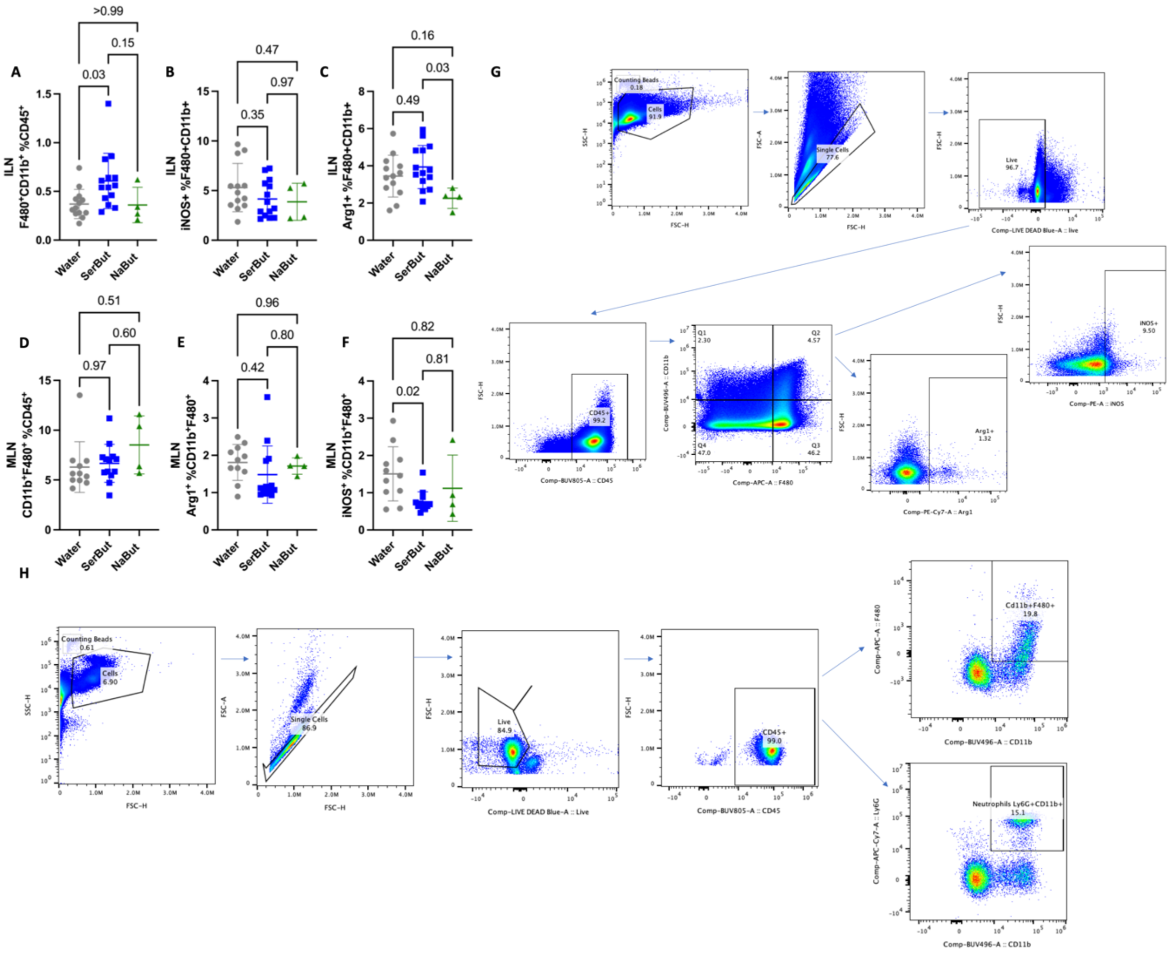

Supplemental Figure 6. Flow cytometry of Macrophage populations in the blood and lymph nodes. A-C. Inguinal lymph node flow cytometry. **A.** CD11b$^+$F480$^+$ macrophages %CD45$^+$. **B.** iNOS$^+$ inflammatory macrophages. **C.** Anti-inflammatory Arg1$^+$ macrophages. **D-F.** mesenteric lymph node flow cytometry. **D.** CD11b$^+$F480$^+$ macrophages %CD45$^+$. **E.** iNOS$^+$ inflammatory macrophages. **F.** Anti-inflammatory Arg1$^+$ macrophages. **H.** Lymph node flow gating strategy. **I.** Blood flow gating strategy. Statistical analyses were performed using a one-way ANOVA with a Tukey's, Welch's (if standard deviations were significantly different by Bartlett and Brown-Forsyth tests), or Kruskal Wallis's (if data were not normally distributed determined by Shapiro-Wilk test) post-test.

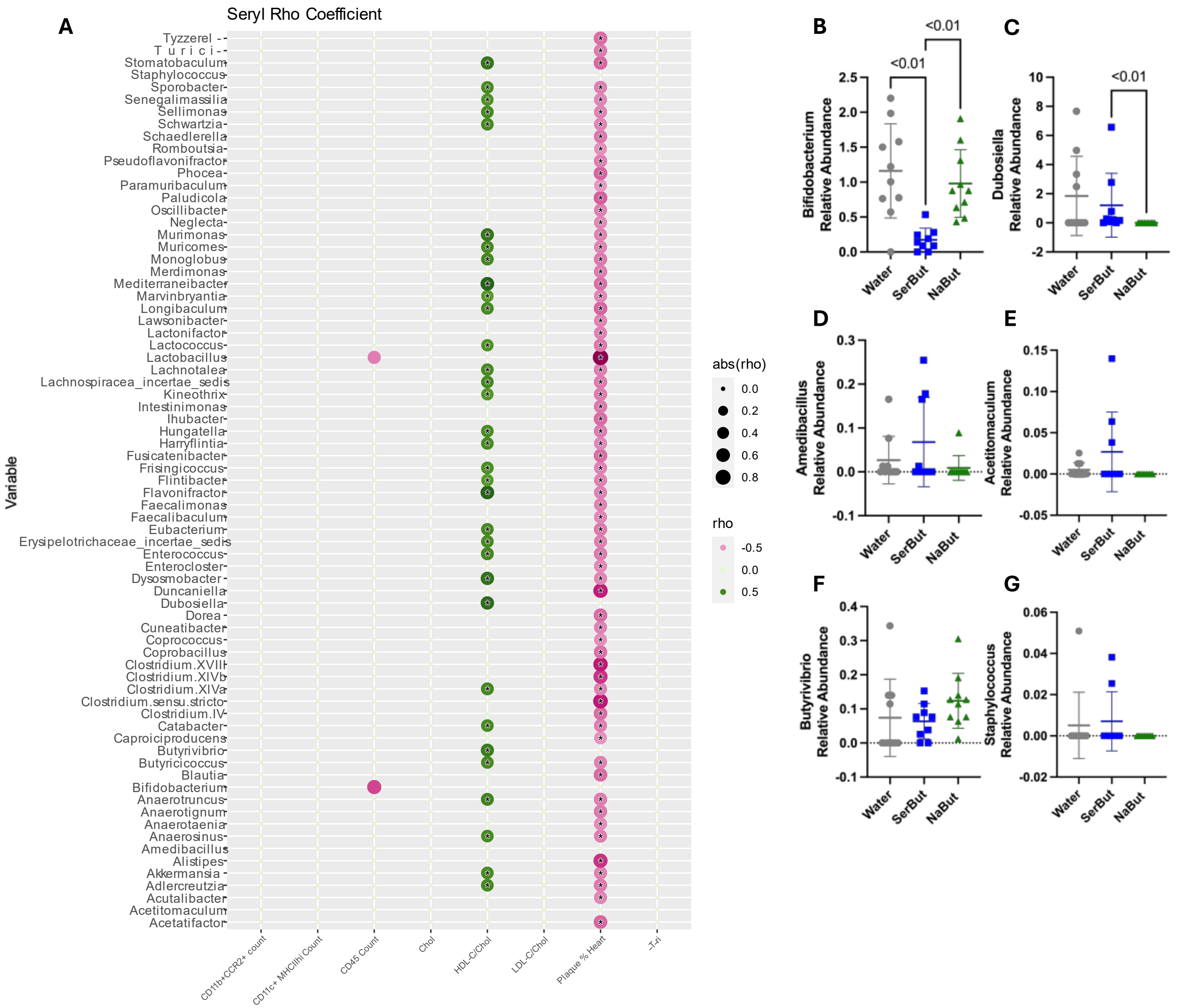

Supplemental Figure 7. Ecosystem-wide and genus-level large-bowel microbiome variation, SerBut, or general butyrate effects on plaque in the heart. **A.** Multiple linear regression displaying all pathology readouts. Associations between plaque in the heart and Seryl modification of butyrate after partialing out the effect of butyrate and genus level relative abundance as covariates. **B-G.** Relative abundance of bacterial genus that returned rho of zero. **B.** Bifidobacterium **C.** Dubosiella **D.** Amedibacillus **E.** Acetitomaculum **F**. Butyrivibrio **G.** Staphylococcus. Positive correlations (between treatment/microbiome factor and pathology factor) are displayed in green as the rho correlation coefficient. Negative correlations are displayed in pink as the rho correlation coefficient. Color intensity is proportional to transformed rho coefficient. Astrix denote significance after multiple testing correction (FRD) on every row value in a specific column (pathologies) using an FDR of 0.05.

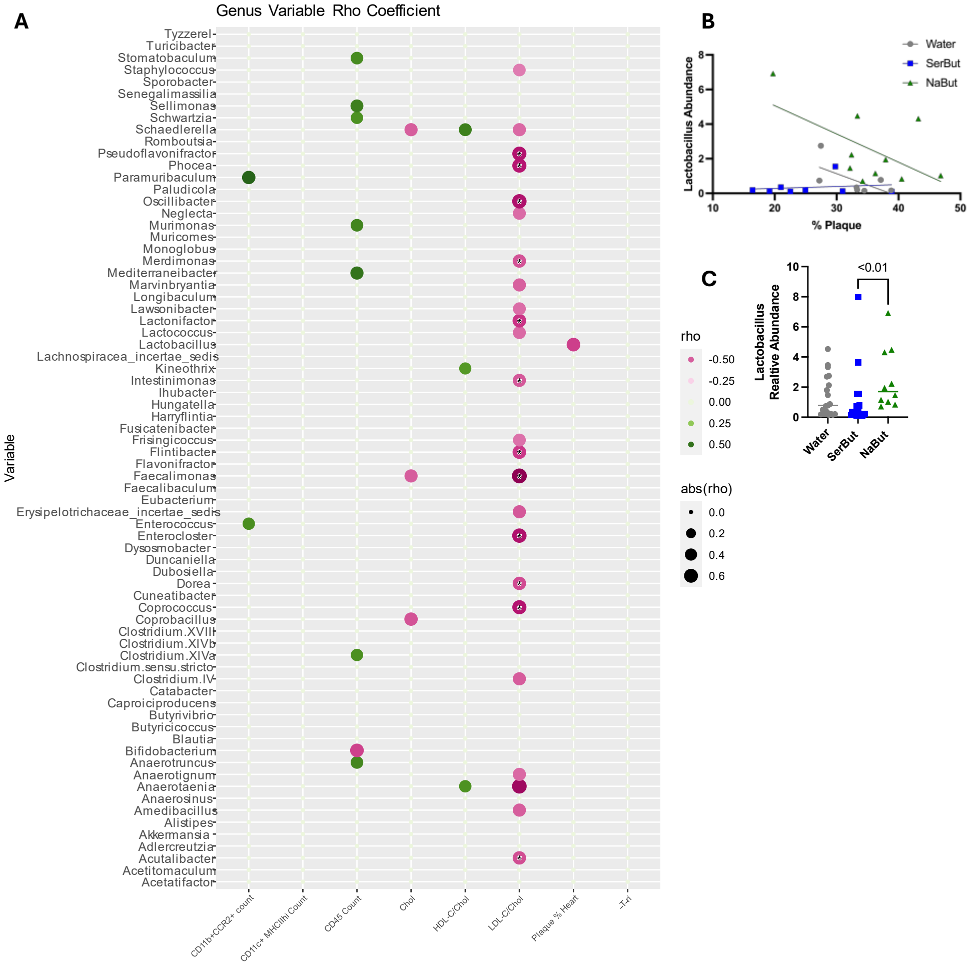

Supplemental Figure 8. Ecosystem-wide and genus-level large-bowel microbiome variation, SerBut, or general butyrate effects on plaque in the heart. **A.** Multiple linear regression displaying all pathology readouts. Associations between plaque in the heart and Genus relative abundance after partialing out the effect of butyrate and seryl modification of butyrate as covariates. **B.** Correlation between Lactobacillus relative abundance and the percent plaque in the aortic root per group. **C.** The relative abundance of Lactobacillus. Positive correlations (between treatment/microbiome factor and pathology factor) are displayed in green as the rho correlation coefficient. Negative correlations are displayed in pink as the rho correlation coefficient. Color intensity is proportional to transformed rho coefficient. Astrix denote significance after multiple testing correction (FRD) on every row value in a specific column (pathologies) using an FDR of 0.05.

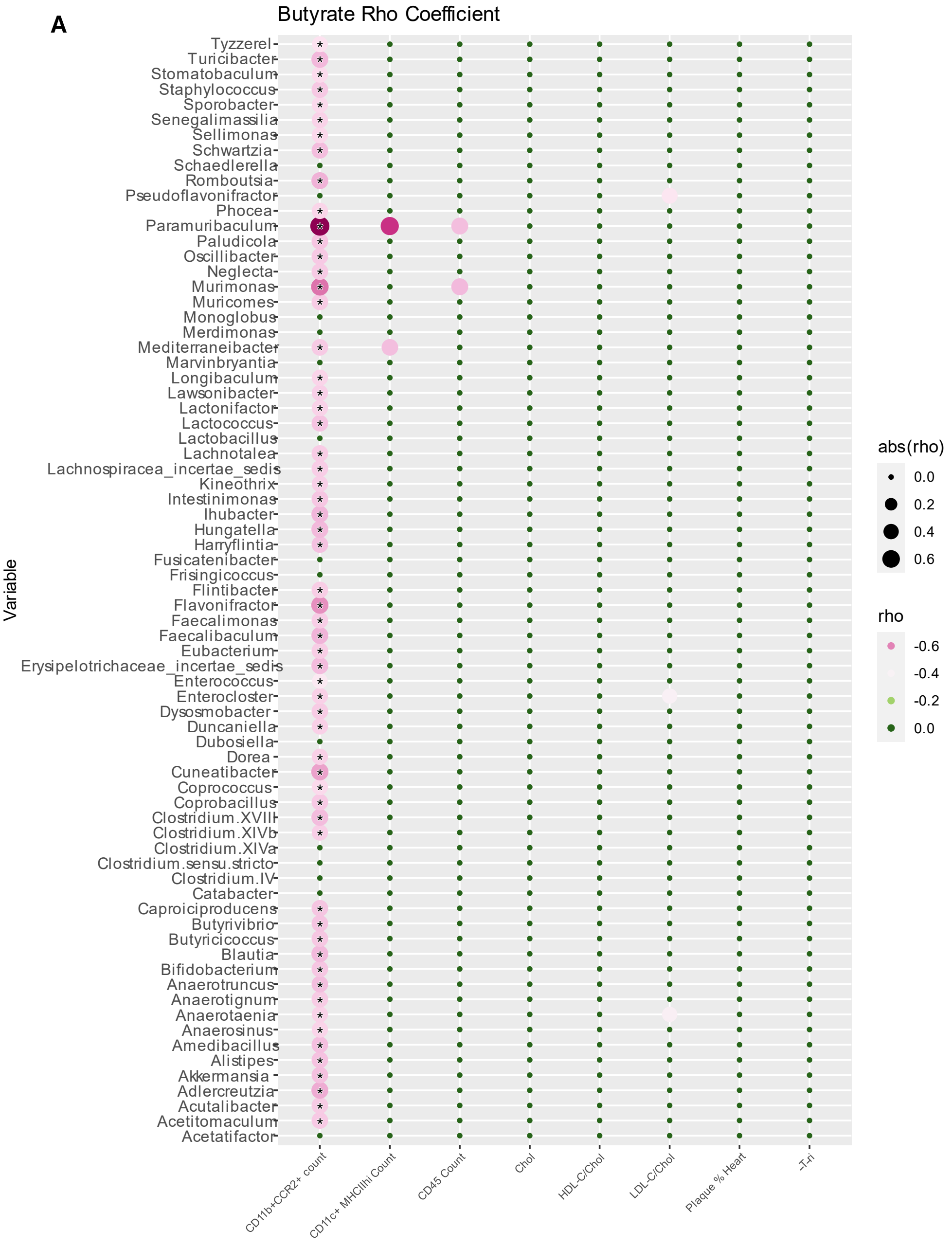

Supplemental Figure 9. Ecosystem-wide and genus-level large-bowel microbiome variation, SerBut, or general butyrate effects on plaque in the heart. **A.** Multiple linear regression displaying all pathology readouts. Associations between plaque in the heart and any form of butyrate after partialing out the effect of genus relative abundance and seryl modification of butyrate as covariates. Positive correlations (between treatment/microbiome factor and pathology factor) are displayed in green as the rho correlation coefficient. Negative correlations are displayed in pink as the rho correlation coefficient. Color intensity is proportional to transformed rho coefficient. Astrix denote significance after multiple testing correction (FRD) on every row value in a specific column (pathologies) using an FDR of 0.05.

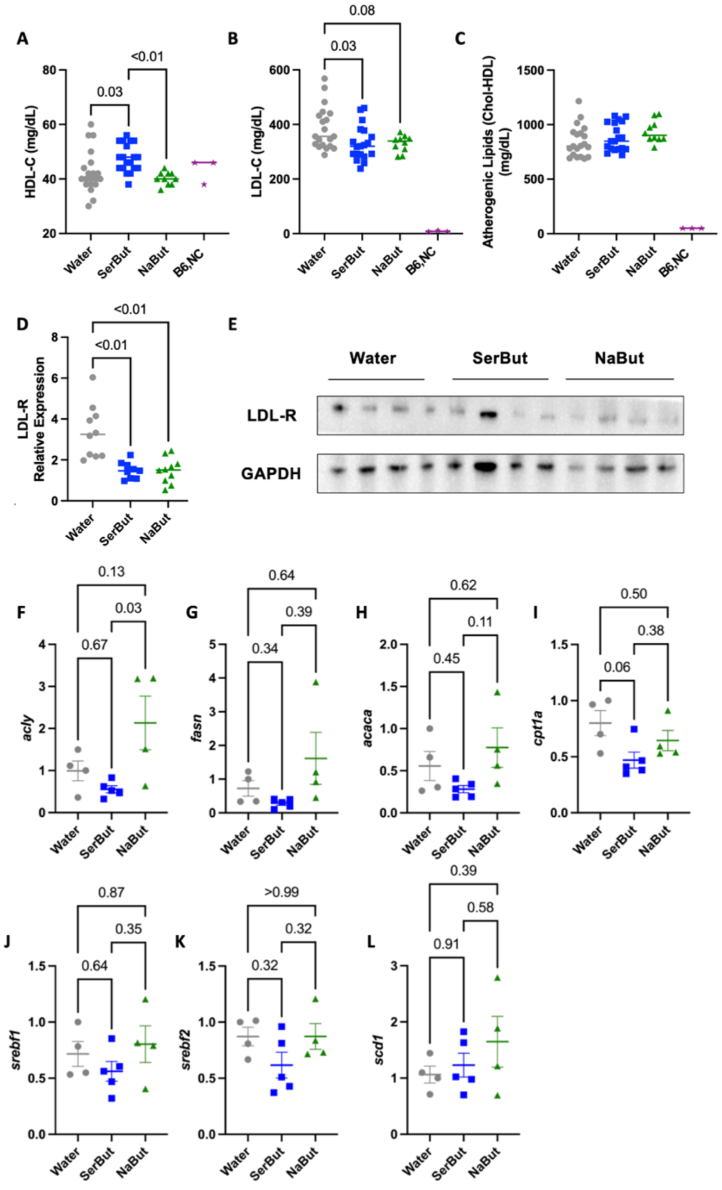

Supplemental Figure 10. A. HDL-C in plasma. **B.** LDL-C in plasma. **C.** Atherogenic Lipids in plasma. Livers of female ApoE$^{-/-}$ mice on a HFD in concert with 150 mM SerBut, NaBut, or water ad libitum for 6 weeks were taken and homogenized for analysis. **D.** Western blot quantification of LDL-R. **E.** Representative Western blot of LDL-R and GAPDH on livers. **F-L.** qPCR analysis of livers taken at 6 weeks. Liver metabolism genes **F**. acly, **G.** fasn. **H.** acaca, **I.** cpt1a, **J.** srebf1, **K.** srebf2, **L.** scd1. Quantification of mean optical density of each Western Blot band was normalized over the mean optical density of the same samples GAPDH band. Statistical analyses were performed using a one-way ANOVA with a Tukey's, Welch's (if standard deviations were significantly different by Bartlett and Brown-Forsyth tests), or Kruskal Wallis's (if data were not normally distributed determined by Shapiro-Wilk test) post-test.
