# Supplementary material for: A prometabolite strategy inhibits cardiometabolic disease in an ApoE–/– murine model of atherosclerosis
Source: JCI Insight. 2025 Aug 8;10(15):e191090. doi: 10.1172/jci.insight.191090 (PMC12333940; doi:10.1172/jci.insight.191090)
Supplement: Unedited blot and gel images [file jciinsight-10-191090-s237.pdf]

Uncropped Unedited Western Blot

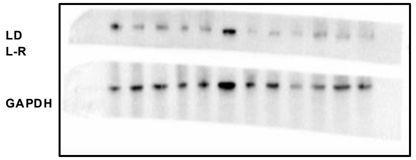

*Membrane was cut based on ladder and stained for each antibody in only the relevant kDa section of the membrane.
